# Bimetallic Nickel–Palladium Nanoparticles Supported on Multiwalled Carbon Nanotubes for Suzuki Cross-Coupling Reactions in Continuous Flow

**DOI:** 10.1021/acs.iecr.4c02922

**Published:** 2025-01-07

**Authors:** Katherine A. Wilson, Harlee B. Winkleman, Ali R. Siamaki

**Affiliations:** Department of Chemistry, Physics, and Materials Science, Fayetteville State University, Fayetteville, North Carolina 28301, United States

## Abstract

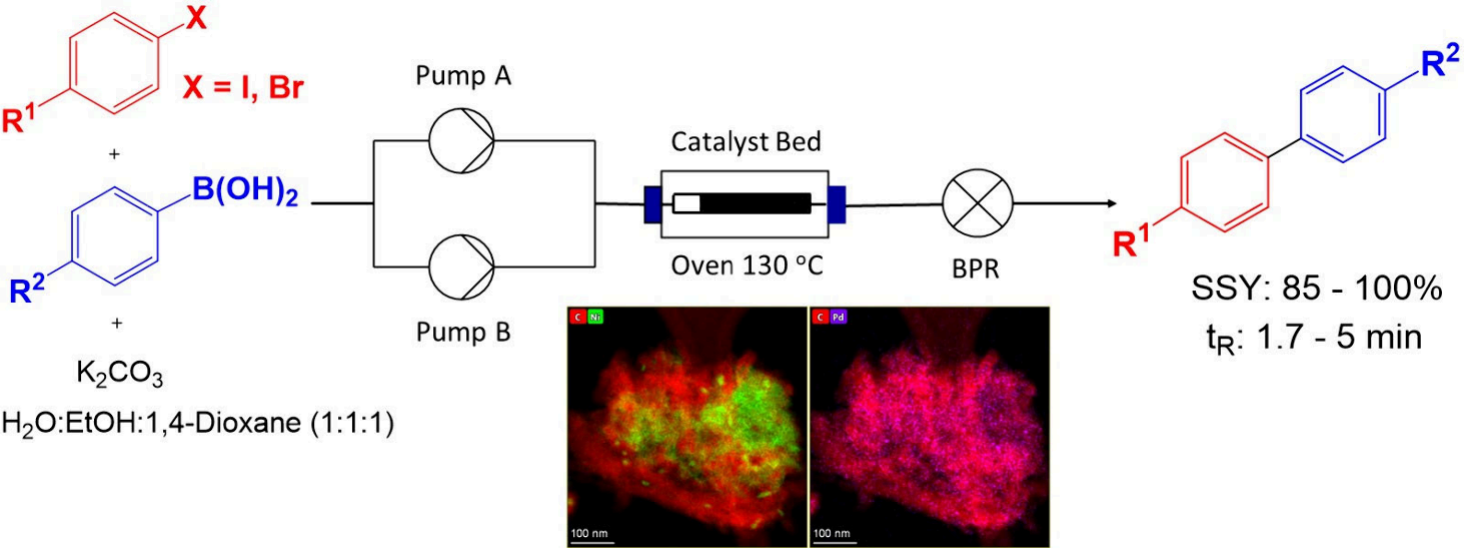

An efficient Suzuki cross-coupling reaction under continuous flow conditions was developed utilizing an immobilized solid supported catalyst consisting of bimetallic nickel–palladium nanoparticles (Ni–Pd/MWCNTs). In this process, the reactants can be continuously pumped into a catalyst bed at a high flow rate of 0.6 mL/min and the temperature of 130 °C while the Suzuki products are recovered in high steady-state yields for prolonged continuous processing. The catalyst was prepared by mechanical shaking of the appropriate nickel and palladium salts using ball-mill energy without the requirement of any solvent or reducing agent. This straightforward, facile, and simple method allows for bulk production of Ni–Pd/MWCNTs nanoparticles with a small particle size ideal for application in continuous flow cross-coupling catalysis. The as-prepared catalyst mostly contains nickel (7.9%) with a very small amount of palladium (0.81%) according to ICP-OES analysis. This remarkable immobilized catalyst can be used several times for different Suzuki reactions with a minimum loss of reactivity and no detectable leaching of the metal nanoparticles. Notably, by modifying the groups on both aryl halides and phenylboronic acids, the method provides access to a diverse array of the Suzuki products in flow with high steady-state yield, making it suitable for applications in industrial and pharmaceutical scales. Moreover, several spectroscopic techniques were employed to identify the structure and composition of the as-prepared Ni–Pd/MWCNTs nanoparticles before and after the reaction in flow such as transmission electron microscopy (TEM), X-ray diffraction (XRD), X-ray photoelectron spectroscopy (XPS), thermogravimetric analysis (TGA), BET surface area (physisorption), and FTIR spectroscopy.

## Introduction

The Suzuki cross-coupling reaction provides a powerful method for the formation of complex organic molecules through the formation of a carbon–carbon bond between the organic halides or triflates and organoboron reagents.^1−4^ The high selectivity and stability of boron reagents in addition to their availability and safety have contributed to this approach, remaining it as an ideal method for the preparation of a vast number of molecules with greater molecular complexity.^5−7^ Thus, the method is widely used for both laboratory small-molecule synthesis and commercial scale operations with considerable attention being given for the preparation of fine chemicals and pharmaceutical active products.^8,9^ These reactions are in most cases carried out in commercial batch processes utilizing a catalyst in the homogeneous form and ligands to enhance both the selectivity and reactivity of the catalyst.^10^ Under batch reaction conditions, the reagents are introduced to the reaction vessel in the presence of the catalyst and heated at a certain temperature and specific time, while the products are recovered over the course of the reaction. However, it would be difficult to effectively take advantage of the elevated catalytic activity under batch reaction conditions given the short reaction times required to affect conversion to products.^11^ While effective due to their greater flexibility and diversity scope, the batch processes have certain inherent limitations such as lower heat and mass transfer rates, greater operation costs, higher safety hazards, and expensive purification steps.^12^ In addition, the use of homogeneous catalysis in this process has implemented a further drawback to the viability of batch reactions due to the major difficulty in removing the catalyst from the reaction mixture, thus increasing the metal contamination in the final product.^13^ The contamination of the metal residue is one of the most important drawbacks toward implementing extensive applications of this method for pharmaceutical purposes.^14,15^ To address this shortcoming, ligand-free heterogeneous palladium catalysis has been developed over the past years as a facile, cost-effective, and robust approach, as has been seen in the significantly growing research in this area.^16−19^

As an alternative approach, continuous flow reactor systems have gained increasing attention in organic synthesis with the prospect of improving heat and mass transfer to expand the limits of traditional batch operating conditions.^20−23^ Considering a greater surface to volume ratio in a flow reactor, the catalyst contact time can be significantly reduced by adjusting an appropriate flow rate and residence time, resulting in more efficient heat transfer.^24−26^ This is particularly true in the general area of catalysis where the large surface-to-volume ratios can have a dramatic effect on the space–time yields.^27−29^ In this context, solid based heterogeneous catalysis in which the metal is fixed on the solid support presents a promising option to address the necessity for an efficient catalytic reaction in flow.^30−32^ The reagents can be pumped into the packed-bed catalyst at a certain temperature and flow rate followed by subsequent collection of the product continuously.

The immobilization and stability of solid supported metal catalyst in the packed-bed column plays a crucial role in determining the residence time, product selectivity, product yield, and the overall throughput.^33−36^ As such, the preparation of effective heterogeneous catalytic systems capable of being immobilized and separated from the reaction solution is an important task that has a considerable impact on cross-coupling applications in continuous flow. This will require strong interactions and binding between the metal and the solid support.

Carbon nanotubes are recently considered as a superior solid support in cross-coupling reactions owing to their incredible thermal stability, extensive surface area, and outstanding electronic structure suitable for stabilizing the metal nanoparticles due to the presence of sp^2^ carbon atoms within the structures.^37,38^ These materials are available in the form of single-walled carbon nanotubes (SWCNTs) with a hollow hexagonal single roll of the graphene or multiwalled (MWCNTs) consisting of multilayered rolled units with several nanometers in diameters (2–25 nm) and many micrometers in length, providing excellent electron conductivity and electrochemical and chemical stability in different media.^39−41^ Of particular importance, these materials exhibit a large surface area (2600 m^2^ g^–1^) with extended π-conjugation within their multilayer’s cylindrical structures, being desirable for anchoring a variety of metallic and bimetallic nanoparticles for heterogeneous catalysis.^42−45^ We have recently demonstrated the remarkable applications of MWCNTs supported metal nanoparticles in Suzuki and Sonogashira coupling reactions in batch.^46−48^ The catalyst was prepared by simple and straightforward solventless mechanochemical shaking of the metal salts and multiwalled carbon nanotubes using a ball-mill mixer without the necessity of any solvent or reducing agent.^49,50^ In this process, a partial decomposition of the precursor materials including metal salts and carbon nanotubes occurs under the mechanical energy of the aggressive ball-milling, leading to the formation of metal nanoparticles with the size of 3–5 nm deposited evenly on the surface of MWCNTs at ambient temperature. This procedure was effectively used for the preparation of MWCNTs supported palladium and nickel nanoparticles (Pd/MWCNTs), bimetallic magnetic Ni–Fe_3_O_4_/MWCNTs, and Ni–Pd/MWCNTs nanoparticles.^47,48^

Nickel is a more abundant and less expensive element and considered as one of the ideal and affordable alternatives to palladium in cross-coupling reactions due to its similar electronic structure, strong binding interaction, and formation of multiple oxidation states.^51−54^ Recently, due to the greater catalytic activity, high selectivity, and excellent stability, the nickel–palladium based bimetallic nanoparticles have attracted considerable attention as the catalyst in a variety of cross-coupling reactions. The addition of the more available and less expensive metal such as nickel can significantly reduce the cost of the catalysis by lowering the amount of palladium in the bimetallic system while still providing great catalytic activity, making the reaction more economically feasible.^55−58^

We have successfully employed the magnetic Ni–Fe_3_O_4_/MWCNTs nanoparticles in Suzuki coupling reactions with excellent recyclability and a great turnover number (19000). Due to the magnetic properties of these materials, the catalyst can be easily removed from the reaction mixture by a strong magnet, washed with an appropriate solvent, and used repeatedly in the next runs.^47^ Similarly, the bimetallic nickel–palladium supported on MWCNTs (Ni–Pd/MWCNTs) exhibited superior application in copper-free Sonogashira cross-coupling reactions in high yield, achieving a great diversity of products from both aryl halides and terminal alkynes. Utilizing more nickel, we have been able to reduce the amount of palladium in this catalyst to as low as 0.81% with no significant effect on reactivity.^48^ It is evident that the addition of the nickel into the palladium in the bimetallic mixture not only significantly reduces the preparation cost but also proves the enhanced catalytic activity in Sonogashira coupling. Likewise, the catalyst can be recycled multiple times, affording a high yield of the product with a great turnover number (TON) of 7200 and turnover frequency of 21600 h^–1^. The outstanding reactivity and high turnover number of Ni–Pd/MWCNTs in batch reactions have prompted us to expand the application of these nanoparticles in continuous flow. The ease of preparation of these materials through a facile, cost-effective, and direct solventless method provides access to a multigram quantity of the catalyst in a short amount of time desirable for the scale-up process. In addition, the use of nickel has a significant impact on the cost of catalysis. We present in this work the highly active and incredibly robust Ni–Pd/MWCNTs nanoparticles to serve as the immobilized solid supported catalyst in Suzuki cross-coupling reactions under continuous flow conditions. This bimetallic system mostly consists of nickel (7.9%) and a very small amount of palladium (0.81%). The catalyst was packed into a highly compact glass column (o.d., 15 mm), and the mixture of reagents was passed through the column using high-pressure pumps. This packed column is designed to provide extremely efficient mixing of chemical reactants and is easily scalable from analytical milligram applications to commercial processes. The optimal flow rate for the catalysis was adjusted to obtain a high conversion of Suzuki products in a short residence time. The Ni–Pd/MWCNTs nanoparticles are extremely robust and can be employed in multiple Suzuki reactions. Alternatively, two packed glass columns can be placed in parallel to increase the production capacity. The catalyst can be prepared easily via a simple, low-cost, and one-step approach without utilizing any solvents, ligands, or reducing agents. Therefore, this approach can be potentially attractive for industrially relevant commercial applications and large-scale operations using continuous throughput manufacturing technologies for carbon–carbon bond forming reactions, making it suitable for economically viable scale up processes with the prospective in chemical and pharmaceutical applications.

## Experimental Section

### General Methods

Multiwalled carbon nanotubes (MWCNTs) of 30–50 nm were purchased from ACS Material, Advanced Chemicals Supplier. Nickel(II) acetate tetrahydrate and palladium(II) acetate were purchased from Sigma-Aldrich. Aryl iodides and bromides, phenylboronic acid, and other substituted boronic acids were obtained from Sigma-Aldrich, Alfa Aesar, and ACROS Organics and used as received. A mixture of deionized water, ethanol, and 1,4-dioxane (1:1:1) was used to dissolve the reagents and pump them through the flow reactor. Shimadzu GC-MS QP2010 SE was used to complete the gas chromatography–mass spectroscopy (GC-MS) analysis of the organic products. Thermo Fisher Talos F200X G2, a 200 kV FEG (field emission gun) at Analytical Instrumentation Facilities at North Carolina State University (AIF) was employed to perform the TEM analysis of the catalysts. Nuclear magnetic resonance spectroscopy measurements, including those of ^1^H and ^13^C NMR, were obtained utilizing a JEOL 400 MHz spectrometer. A Uniqsis FlowLab reactor equipped with high-pressure pumps, a hotcoil, and a glass column of 10 mm i.d., (15 mm o.d.) PTFE/PEEK with tubing, being adjustable, was used for continuous flow reactions. A Kratos Axis Supra X-ray photoelectron spectrometer at University of North Carolina—Chapel Hill (UNC—Chapel Hill) instrumentation facilities (CHANL) was used to complete the XPS analysis. Inductively coupled plasma-optical emission spectroscopy (ICP-OES) analysis was completed using iCAP 7200 (Thermo Fisher Scientific). A Rigaku MiniFlex 600 X-ray diffractometer (XRD) was used to conduct the XRD analysis of the solid catalysts before and after the flow reaction. Thermal gravimetric analyzer Shimadzu DTG-60A was employed for TGA/DTA analysis. An Anton Paar high-vacuum physisorption analyzer Autosorb iQ was used for the BET isotherm surface area and pore size measurements. A Thermo Fisher Scientific Nicolet iS50 FTIR spectrometer was used for FTIR analysis of the samples.

### Synthesis of Solid Supported Catalysts

The following compositions were prepared and screened to investigate the best catalytic system for Suzuki reactions in flow. The previously published procedure^48^ was used with slight modification in concentration of the metals to prepare the catalysts. Each sample was placed in zirconium ceramic vials and subjected to extensive mechanical shaking for 45 min at ambient temperature using a SPEX 8000 M ball-mill mixer. The mechanical movement of the ball-mill provided 1060 cycles per minute with 5.9 cm back and forth and 2.5 cm side to side. The following concentrations were prepared:

#### Ni–Pd/MWCNTs (10% Ni, 1% Pd)

Nickel acetate tetrahydrate (42.39 mg, 10% Ni content) and palladium acetate (2.11 mg, 1% Pd content) were mixed with multiwalled carbon nanotubes (MWCNTs) (89 mg).

#### Ni/MWCNTs: (20% Ni)

Nickel acetate tetrahydrate (84.78 mg, 20% Ni content) was mixed with MWCNTs (80 mg).

#### Ni/MWCNTs: (10% Ni)

Nickel acetate tetrahydrate (42.39 mg, 10% Ni content) was added to MWCNTs (80 mg).

#### Pd/MWCNTs: (1% Pd)

Palladium acetate (2.11 mg, 1% Pd content) was added to MWCNTs (99 mg).

### General Procedure for Continuous Flow Suzuki Cross-Coupling Reactions

The continuous reaction was performed using a Uniqsis FlowLab reactor equipped with two high-pressure pumps, a hot coil oven, and an Omnifit glass column in which the solid supported catalyst can be introduced and packed. A solution of 4-iodobenzaldehyde (300 mg, 1.29 mmol), phenylboronic acid (188.7 mg, 1.55 mmol), and potassium carbonate (534 mg, 3.87 mmol) in a mixture of H_2_O:EtOH:1,4-dioxane (150 mL) was pumped through the reactor column (Omnifit, 15 mm o.d., 10 cm length) packed with 300 mg of the corresponding catalyst at a specific flow rate while maintaining a reaction temperature of 130 °C using a hotcoil oven. The flow rate was adjusted accordingly to maintain a high steady-state yield. The product was collected in fractions (5–10 mL), and each fraction was analyzed by GC-MS spectroscopy. For prolonged on-stream reaction in Figure 3, 4-iodobenzaldehyde (2.4 g, 10.3 mmol), phenylboronic acid (1.5 g, 12.4 mmol), and potassium carbonate (4.3 g, 31.02 mmol) in a mixture of H_2_O:EtOH:1,4-dioxane (1.2 L) were passed through the reactor column (Omnifit, 15 mm o.d., 10 cm length) packed with 300 mg of the corresponding catalyst at a specific flow rate while maintaining a reaction temperature of 130 °C using a hotcoil reactor. The reaction mixtures were collected in fractions and analyzed by GC-MS spectroscopy. The fractions were combined and extracted with dichloromethane (2 × 5 mL). The organic layers were decanted, dried over sodium sulfate, and filtered. After removal of the solvent under reduced pressure, the final product was subjected to flash chromatography using hexane:ethyl acetate.

### Procedure for Continuous Flow Suzuki Cross-Coupling Reaction Using Functionalized Substrates

A solution of aryl halides (1.3 mmol, 1 equiv), aryl boronic acids (1.56 mmol, 1.2 equiv), and potassium carbonate (3.9 mmol, 3 equiv) in a mixture of H_2_O:EtOH:1,4-dioxane (300 mL) was pumped through the reactor column (Omnifit, 15 mm o.d., 10 cm length) packed with 300 mg of the corresponding catalyst at a specific flow rate while maintaining a reaction temperature of 130 °C using a hotcoil oven. The flow rate was adjusted accordingly to maintain a high steady-state yield. The product was collected in fractions (5–10 mL), and each fraction was analyzed using GC-MS technique. For the recovery of the final products, all of the fractions were combined and extracted using dichloromethane (2 × 5 mL). The organic layers were decanted, dried over sodium sulfate, and filtered. The final product was purified by flash chromatography on silica gel using hexane:ethyl acetate as the solvent.

## Results and Discussion

**Scheme 1:**
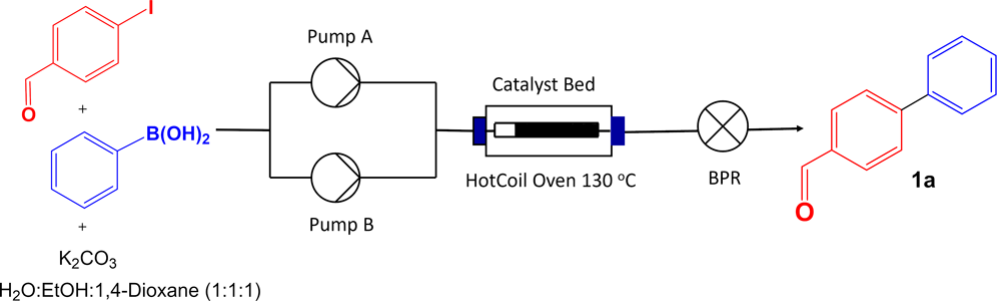
Continuous Flow Suzuki Cross-Coupling Reaction

To investigate the most effective catalyst for the Suzuki reaction in continuous flow, four different catalytic systems were prepared. The overall goal of this effort was to develop an efficient continuous process methodology that would complement and leverage the efficiency observed under batch reaction conditions. In practice, the continuous reaction was performed using the Uniqsis FlowLab reactor equipped with two high-pressure pumps, a hotcoil oven, and an Omnifit glass column in which the solid supported catalyst can be introduced and packed. As shown in Scheme 1, 4-iodobenzaldehyde, phenylboronic acid, and potassium carbonate were dissolved in H_2_O:EtOH:1,4-dioxane (1:1:1) and the mixture was pumped through the reactor column (Omnifit, 15 mm o.d., 10 cm length) packed with 300 mg of the corresponding catalyst in Table 1 at a specific flow rate while maintaining a reaction temperature of 130 °C using a hotcoil oven. The presence of 1,4-dioxane is necessary to retain the solubility of the reagents and product through the flow system. Inductively coupled plasma-optical emission spectroscopy (ICP-OES) was employed to measure the actual metal content in each catalyst as shown in Table 1.

**Table 1:** Composition of Catalysts Determined by ICP-OES^a^

Catalyst	Ni (wt %)	Pd (wt %)	MWCNTs (wt %)
Ni/MWCNTs	10(8.2)	0	90
Ni/MWCNTs	20(18.5)	0	80
Ni–Pd/MWCNTs	10(7.9)	1(0.81)	89
Pd/MWCNTs	0	1(0.86)	99

a Concentrations in parentheses were determined by ICP-OES.

**Figure 1:**
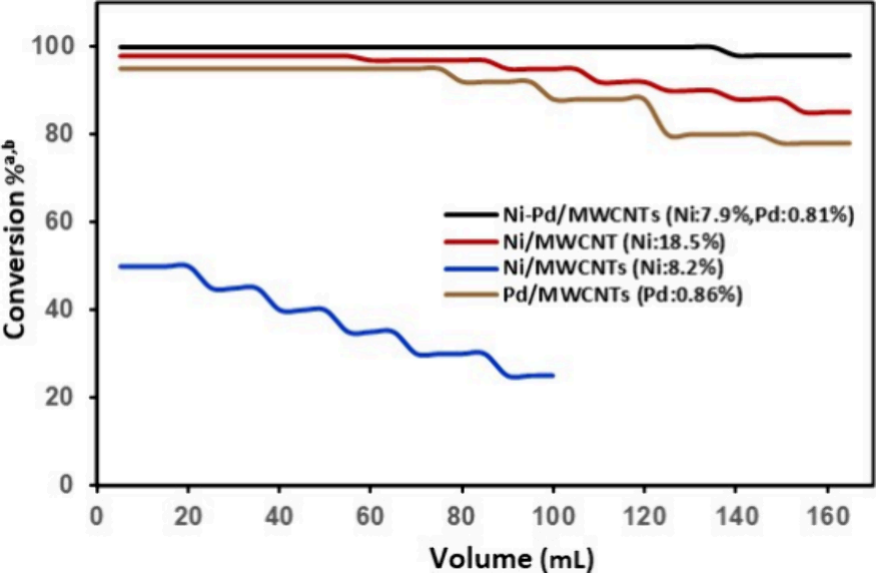
Screening the catalyst for the Suzuki cross-coupling reaction in flow. (a) Reaction conditions: 4-iodobenzaldehyde (300 mg, 1.29 mmol), phenylboronic acid (188.7 mg, 1.55 mmol), and potassium carbonate (534 mg, 3.87 mmol) in a mixture of H_2_O:EtOH:1,4-dioxane (150 mL) passed through the reactor column (Omnifit, 15 mm o.d., 10 cm length) packed with 300 mg of the corresponding catalyst at a specific flow rate while maintaining a reaction temperature of 130 °C using a hotcoil reactor; (b) conversion % determined by GC-MS.

Figure 1 illustrates the conversion percent versus the collected volume of the product (mL) for the Suzuki cross-coupling reaction of 4-iodobenzaldehyde and phenylboronic acid (Scheme 1) at the specific flow rate of 0.2 mL/min for 4 different catalytic compositions in Table 1. The concentration of the product (**1a**) was determined in each collected solution, and the catalyst deactivation was measured using the GC-MS analysis. As shown, the Ni/MWCNTs (Ni, 8.2%) nanoparticles demonstrated extremely low catalytic activity in the flow with conversion of about 50% with a rapid decrease in conversion as the reaction continues. Increasing the amount of nickel in the catalyst proved to have some influence. With 18.5% nickel content (Ni/MWCNTs, Ni: 18.5%), the catalytic activity improved with a high conversion of 95%. Under the continuous flow with 0.2 mL/min flow rate, we have been able to collect 75 mL (105 mg, 0.6 mmol) of Suzuki product at high conversion of 95% before slow deactivation of the catalyst. The conversion dropped further at 125 mL to about 85%. Alternatively, Ni–Pd/MWCNTs (Ni, 7.9%; Pd, 0.81%) exhibited excellent catalytic activity with remarkably prolonged reactivity. When loading this catalyst in the packed glass column, we obtained a quantitative conversion of 100% for an extended collection of 150 mL (230 mg, 1.26 mmol) of the corresponding biphenyl product at a flow rate of 0.2 mL/min. Ni–Pd/MWCNTs continued to show a quantitative conversion % even above 160 mL of collection. This great reactivity has been evident for the bimetallic combination of the nickel–palladium catalytic system perhaps due to an electronic interaction between Pd and Ni.^48,59−62^ Notably, Pd/MWCNT nanoparticles containing only 0.86% palladium concentration with no nickel afforded a lower reactivity of 95% for only 75 mL collection (105 mg, 0.6 mmol), and the catalytic activity was slowly diminished after 75 mL (Figure 1). It should be noted that the actual volume of the packed catalyst loaded in the glass column (15 o.d., 30 cm) was 1.0–1.2 mL, which corresponds to 300 mg of the loaded catalyst. The pressure of the system including the glass column was monitored during the entire reaction. The pressure was 12–14 bar when the pure solvent (H_2_O:EtOH:1,4-dioxane, 1:1:1) was purged into the line for wash. The pressure of the packed column during the Suzuki cross-coupling reaction was dropped to 6–8 bar and remained steady during the entire flow process.

**Figure 2:**
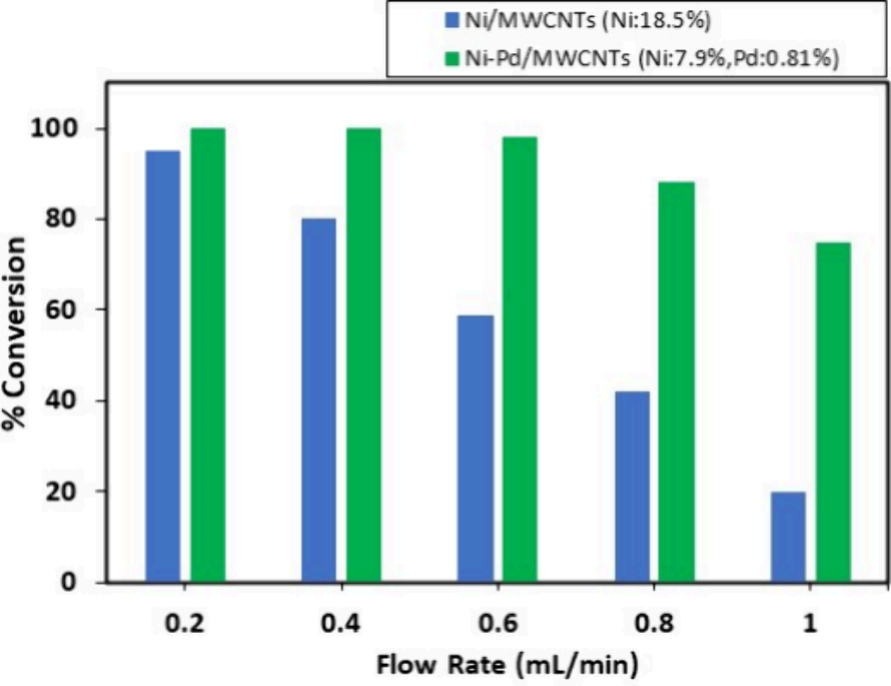
Optimal flow rate for Suzuki cross-coupling reaction in continuous flow.

The optimal flow rate for Suzuki coupling reactions in flow was investigated for Ni/MWCNTs (Ni, 18.5%) and Ni–Pd/MWCNTs (Ni, 7.9%; Pd, 0.81%) (Figure 2). As depicted in Figure 2, both catalysts exhibited high reactivity of 95% and above at 0.2 mL/min. At a higher flow rate of 0.4 mL/min, Ni/MWCNTs nanoparticles revealed a lower conversion of 80% while Ni–Pd/MWCNTs nanoparticles still gave a high conversion of 100% at this flow rate. Increasing the flow rate to 0.6 mL/min diminished the conversion % for Ni/MWCNTs to 59% but had no significant effect on the conversion % using Ni–Pd/MWCNTs. The conversion % for Ni–Pd/MWCNTs nanoparticles slowly drops when the flow rate was increased to 0.7 and 1 mL/min to 88% and 75%, respectively.

To investigate the lifetime and stability of Ni–Pd/MWCNTs (Ni, 7.9%; Pd, 0.81%) nanoparticles in the packed column under the flow conditions, we conducted a prolonged on-stream run of the reference Suzuki cross-coupling reaction in Scheme 1. We used 300 mg of bimetallic Ni–Pd/MWCNTs (Ni, 7.9%; Pd, 0.81%) for this purpose due to superior catalytic performance under continuous flow conditions. In practice, a solution of 4-iodobenzaldehyde, phenylboronic acid, and potassium carbonate in H_2_O:EtOH:1,4-dioxane (1:1:1) were pumped through the packed glass column loaded with 300 mg of the corresponding catalyst. The column was heated at 130 °C, and the product was collected in fractions and monitored using GC-MS. The flow rate of the reaction mixture was controlled by the pump and adjusted incrementally to 0.6 mL/min until the maximum conversion of 100% was established. When the flow rate was adjusted to 0.6 mL/min, a residence time (*t*_R_) of 1.7 min was achieved. Utilizing Ni–Pd/MWCNTs nanoparticles as the catalyst, the Suzuki product (1.85 g, 10 mmol) was continuously obtained over 33 h run time at steady-state yield of 98%. Under these conditions, a reactor throughput of 0.31 mmol h^–1^ was accomplished with a space–time yield of 307 mmol L^–1^ h^–1^ (Table 2), affording a turnover number (TON) of 345 and turnover frequency (TOF) of 10.5 h^–1^.

**Table 2:** Prolong On-Stream Continuous Flow Suzuki Cross-Coupling Reaction^a^

Catalyst^b^	Flow Rate (mL/min)	Run Time (h)	SSY%^c^	*t*_R_ (min)	Throughput (mmol h^–1^)	STY^d^ (mmol L^–1^ h^–1^)
Ni–Pd/MWCNTs	0.6	33	98	1.7	0.3	307
Ni/MWCNTs	0.2	25	90	5	0.1	92

a Reaction conditions: 4-iodobenzaldehyde (2.4 g, 10.3 mmol), phenylboronic acid (1.5 g, 12.4 mmol), and potassium carbonate (4.3 g, 31.02 mmol) in a mixture of H_2_O:EtOH:1,4-dioxane (1.2 L) were passed through the reactor column (Omnifit, 15 mm o.d., 10 cm length) packed with 300 mg of the corresponding catalyst at a specific flow rate while maintaining a reaction temperature of 130 °C using a hotcoil reactor.

b The concentration of metals was determined by ICP-OES as Ni–Pd/MWCNTs (Ni, 7.9%; Pd, 0.81%) and Ni/MWCNTs (Ni, 18.5%)

c The steady state yield (SSY) % was determined by GC-MS.

d Space–time yield (STY) was measured based on the volume of the solid catalyst in the glass column.

**Figure 3:**
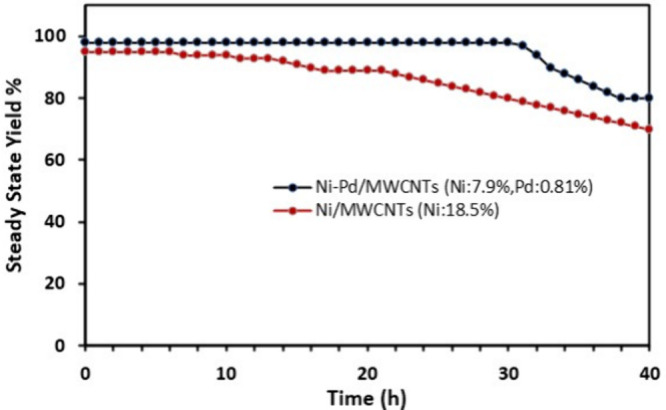
Comparison of bimetallic Ni–Pd/MWCNTs (Ni, 7.9%; Pd, 0.81%) and Ni/MWCNTs (Ni, 18.5%) in flow.

Likewise, the Ni/MWCNTs (Ni, 18.5%) nanoparticles were employed under continuous flow operation, albeit demonstrating a lower reactivity and longer residence time compared to Ni–Pd/MWCNTs nanoparticles. With 300 mg of Ni/MWCNTs nanoparticles loaded in the glass column at the flow rate of 0.2 mL/min, a steady-state yield of 90% was obtained, affording (424 mg, 2.32 mmol) of product over 25 h run time. This afforded a reactor throughput of 0.1 mmol h^–1^ and space–time yield of 92 mmol L^–1^ h^–1^. Interestingly, the analysis of the reaction product from the outlet of the column showed a negligible leaching of the metals after several hours with residual concentrations of nickel and palladium being less than 50 ppb, according to the ICP-OES data. Similarly, the metal content in the reaction solution was analyzed by opening the inlet of the glass column during the flow reaction and taking a sample of the solution from the inlet and middle portion of the column for ICP-OES analysis to determine the metal leaching. The concentrations of nickel and palladium were identified to be less than 100 ppb level in the solution. While both catalysts demonstrated remarkable reactivity for Suzuki cross-coupling reaction in flow, the bimetallic Ni–Pd/MWCNTs proved to be a more efficient and reliable system, providing a higher throughput and shorter residence time with high steady state yield of 98% (Figure 3).

**Figure 4:**
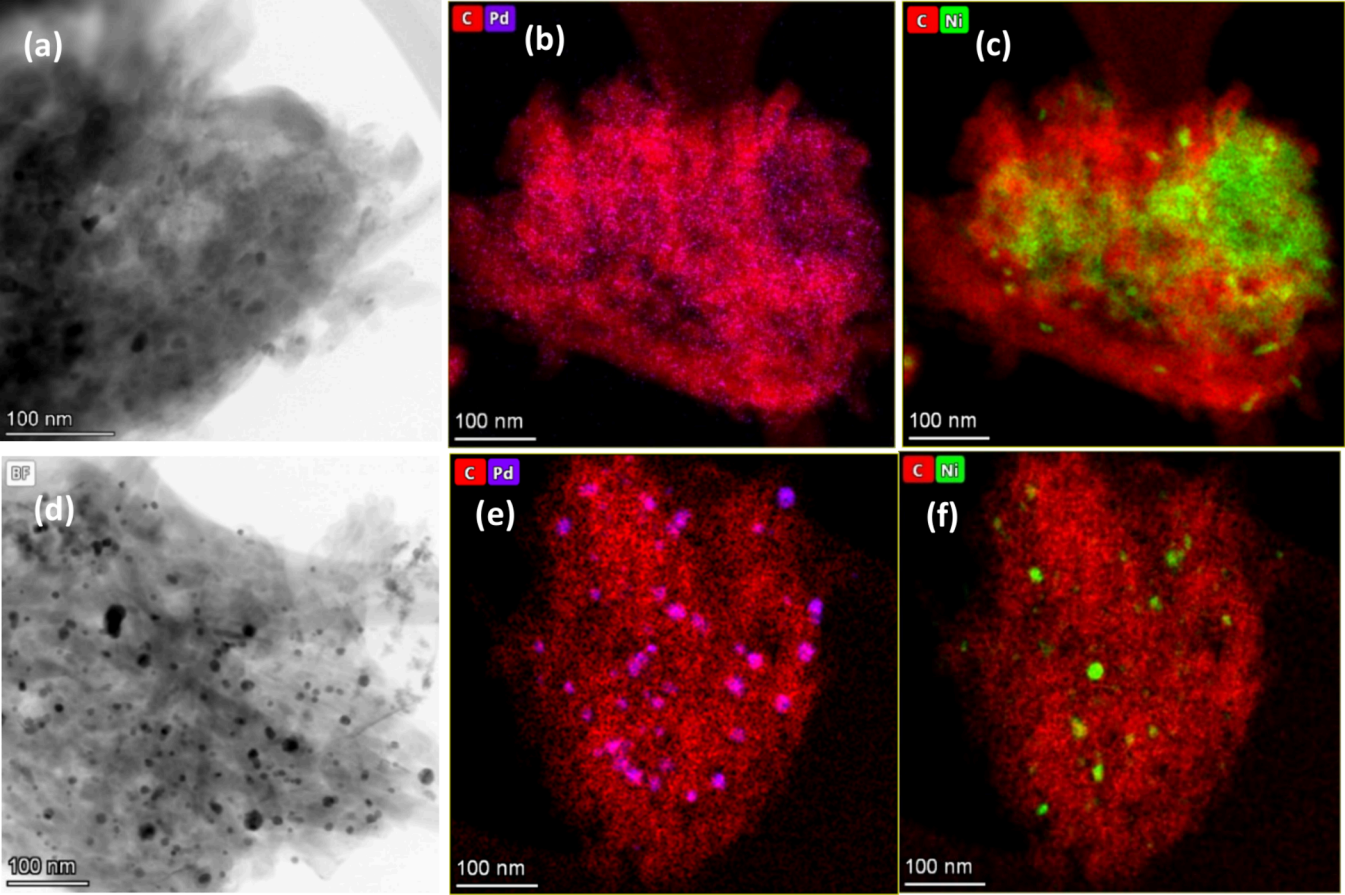
TEM images and EDS mapping: (a–c) Ni–Pd/MWCNTs nanoparticles before the flow reaction; (d–f) Ni–Pd/MWCNTs after the flow reaction.

The structure and composition of the as-prepared Ni–Pd/MWCNTs (Ni:7.9%, Pd:0.81%) nanoparticles were investigated before and after the continuous flow Suzuki reactions by means of transmission electron microscopy (TEM). As revealed from this analysis (Figure 4), both nickel and palladium nanoparticles are evenly deposited on the surface of multiwalled carbon nanotubes in the original sample before the flow reaction in accordance with our previous TEM data for this system.^48^ The average particle size for palladium nanoparticles is between 1 and 5 nm in diameter with a major concentration of the particles on the surface of the nanotubes (Figure 4a,b). This high dispersion and small particle size evidenced a severe effective crushing of the metal salts and MWCNTs under the mechanical energy of the ball-mill to yield a uniform composition of the materials. Nickel nanoparticles are between 10 and 30 nm in diameter and often elongated since they are trapped in inner layers of the MWCNTs (Figure 4c).

Notably, evidence for deactivation of Ni–Pd/MWCNTs nanoparticles was obtained by TEM analysis of this catalyst after prolonged on-stream continuous flow Suzuki reactions (33 h run time). As depicted in Figure 4d–f, the catalyst demonstrated a completely inhomogeneous structure after continuous run time for 33 h. The Pd has clearly clustered into faceted particles. The size of the faceted Pd particles ranges from 10 to 50 nm in diameter. The Ni particle size is also roughly 10–50 nm in diameter. It appears to be far more Pd than Ni on average, with nickel particles segregating into more dense particles. It is obvious from these results that significant agglomeration and accumulation of Pd and Ni nanoparticles are mainly responsible for decreasing the surface area and saturating the coordination sites of the metals, leading to potential deactivation of the catalyst. The TEM analysis of Ni/MWCNTs (18.5%) demonstrated a large cluster of nickel oxide along with some metallic nickel with the particle size of 10–30 nm interspersed with MWCNTs (Supporting Information Figure S1). After the flow reaction was completed (25 h run time), formation of the nickel aggregates to 50 nm particle size was observed by TEM, indicating the potential deactivation of the catalyst (Figure S1).

**Figure 5:**
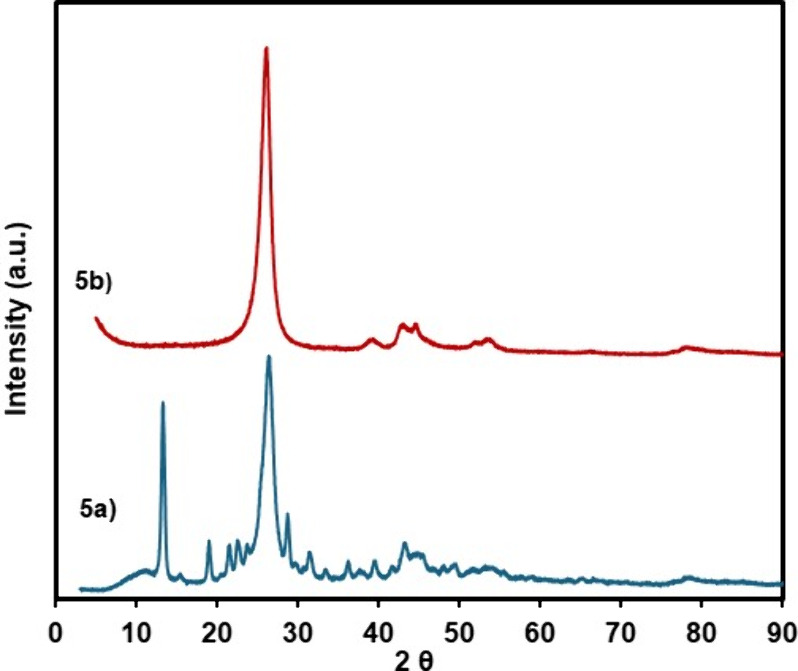
X-ray diffraction (XRD) pattern of Ni–Pd/MWCNTs (a) before the flow reaction and (b) after the flow reaction.

The structure of the Ni–Pd/MWCNTs nanoparticles was further analyzed by XRD to reveal the composition of the catalyst before and after the flow reaction in Scheme 1. As appears in Figure 5a, the Ni–Pd/MWCNTs nanoparticles before the reaction exhibited a crystalline structure due to the strong diffraction pattern observed in these materials.^48^ The nickel nanoparticles demonstrated the corresponding peaks at 45.6°, 54.3°, and 79.2°, which indicate the presence of (111), (200), and (220) face-centered cubic (FCC) of nickel pattern. The observed broad peaks in these diffraction patterns indicated the presence of a smaller crystalline size of nanoscale in this composition. Notably, due to the partial decomposition of the original nickel salts during the ball-mill process, the presence of the nickel acetate is detected as some residue in the background pattern of the XRD spectra. The broad peak at 26.6° is the characteristic diffraction of the (002) peak for the MWCNTs. Due to the small palladium composition in this sample (0.81%), the corresponding peaks for palladium illustrate much lower intensities. The weak signal at about 40.1° can be assigned to *d*-spacing for the (111) plane of metallic palladium, normally observed at around 2.2 Å. The XRD pattern for Ni–Pd/MWCNTs after the continuous flow reaction mainly displays the characteristic signature peaks of nickel nanoparticles at 45.6°, 54.3°, and 79.2° in addition to the strong reflection of MWCNTs at 26.6° (Figure 5b).

**Figure 6:**
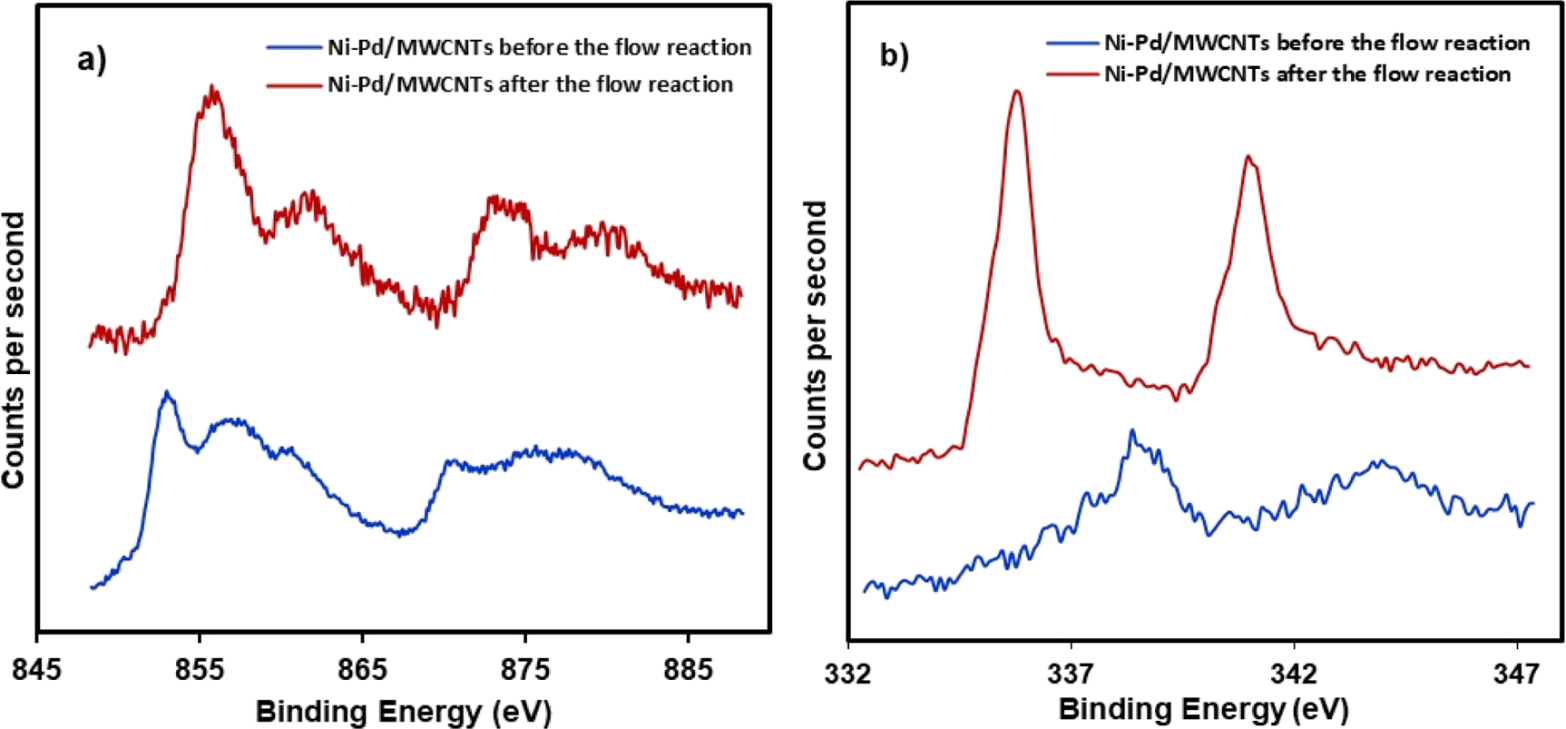
XPS spectra of Ni–Pd/MWCNTs: (a) Ni binding energies and (b) Pd binding energies.

To further evaluate the elemental composition of the metals on the surface of multiwalled carbon nanotubes including the potential oxidation states of the metals, the XPS data of the catalyst were obtained before and after the flow reaction. The presence of nickel, palladium, oxygen, and carbon was initially identified from the survey analysis. As shown in Figure 6a for Ni–Pd/MWCNTs before the flow reaction, the nickel spectrum shows two major peaks around 853 eV (2p_3/2_) and 871 eV (2p_1/2_) for the presence of more metallic Ni 2p_3/2_ in this catalyst, indicating the ball-mill energy causes the nickel to be reduced to form Ni(0), albeit in a very small amount. After the flow reaction, however, a small shift to higher binding energy was observed for Ni 2p with the peaks at 856 eV (2p_3/2_) and 873 eV (2p_1/2_), revealing the formation of Ni(II) perhaps in the form of nickel oxide during the flow reaction. Figure 6b reveals the presence of Pd(II) in the catalyst before the reaction owing to the observed binding energies of Pd 3d_5/2_ measured at 338.6 eV and Pd_3/2_ at 343.7 eV. However, after the flow reaction, the corresponding peaks for palladium have shifted to lower binding energies for Pd 3d_5/2_ and 3d_3/2_ electrons at 335.7 and 341 eV, respectively. We believe that this change could be due to an in situ reduction of Pd most likely by solvent under the basic condition during the prolonged continuous flow process (33 h run time). In addition, a close interaction can be envisaged as a direct electron transfer between two metals which improve the electronic properties of palladium for catalysis as suggested in the reported studies. Adding nickel to palladium has proved to demonstrate an enhanced catalytic activity.^48,59−62^ Such an interaction can affect the binding energies for palladium by producing more Pd(0) on the surface of MWCNTs, increasing the reactivity of Ni–Pd/MWCNTs nanoparticles during the flow process.

**Figure 7:**
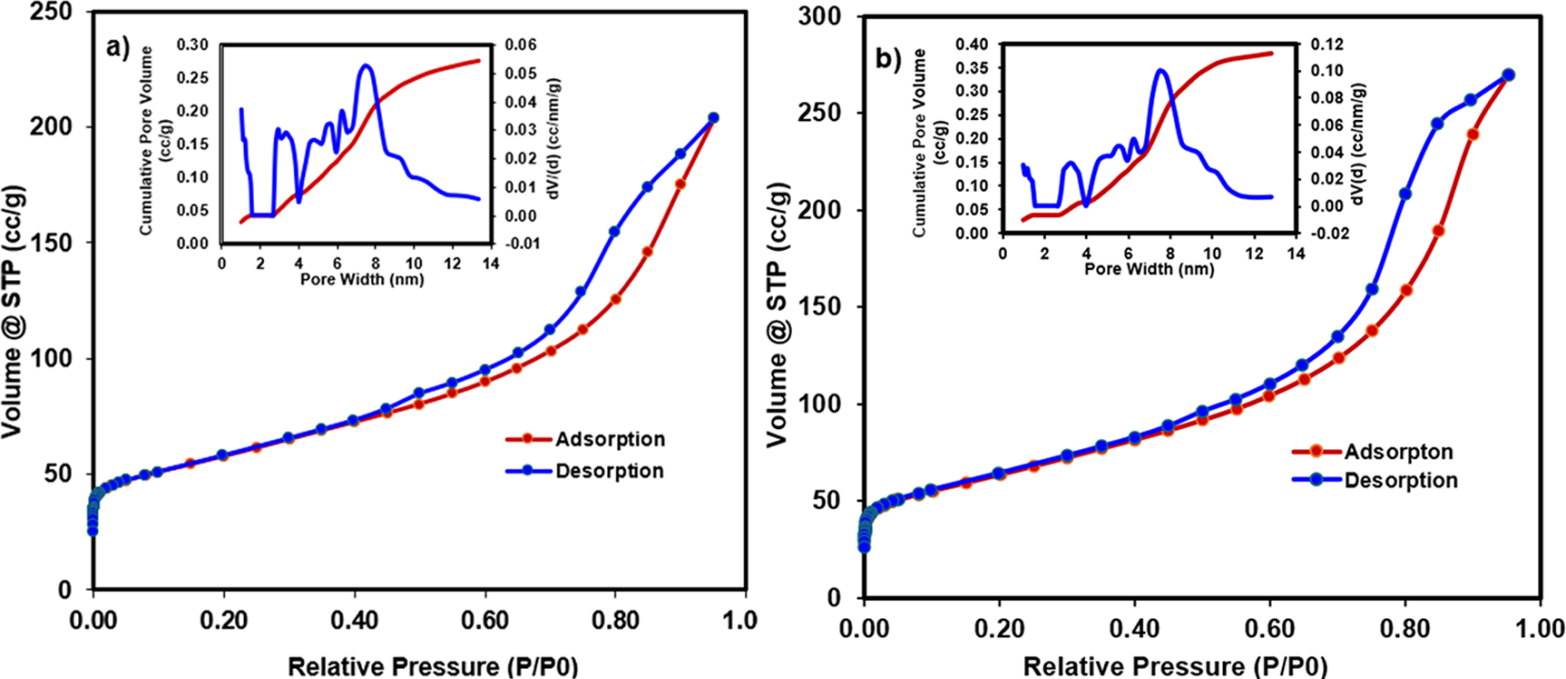
BET analysis of Ni–Pd/MWCNTs (a) before the flow reaction and (b) after the flow reaction.

Figure 7 shows the BET (Brunauer–Emmett–Teller) surface areas and pore size measurements of Ni–Pd/MWCNTs before and after the Suzuki cross-coupling reaction in flow. The adsorption/desorption analysis of the catalyst before the reaction confirmed the surface area of 192.285 m^2^/g and a pore size average of 7.452 nm (Figure 7a). The analysis of the catalyst after the flow reaction revealed no significant changes in the surface area and pore size of the catalyst over a prolonged flow process with a surface area of 203.708 m^2^/g and exactly the same pore size average of 7.452 nm (Figure 7b), indicating that the physical structure of the catalyst remained intact during the catalysis.

**Figure 8:**
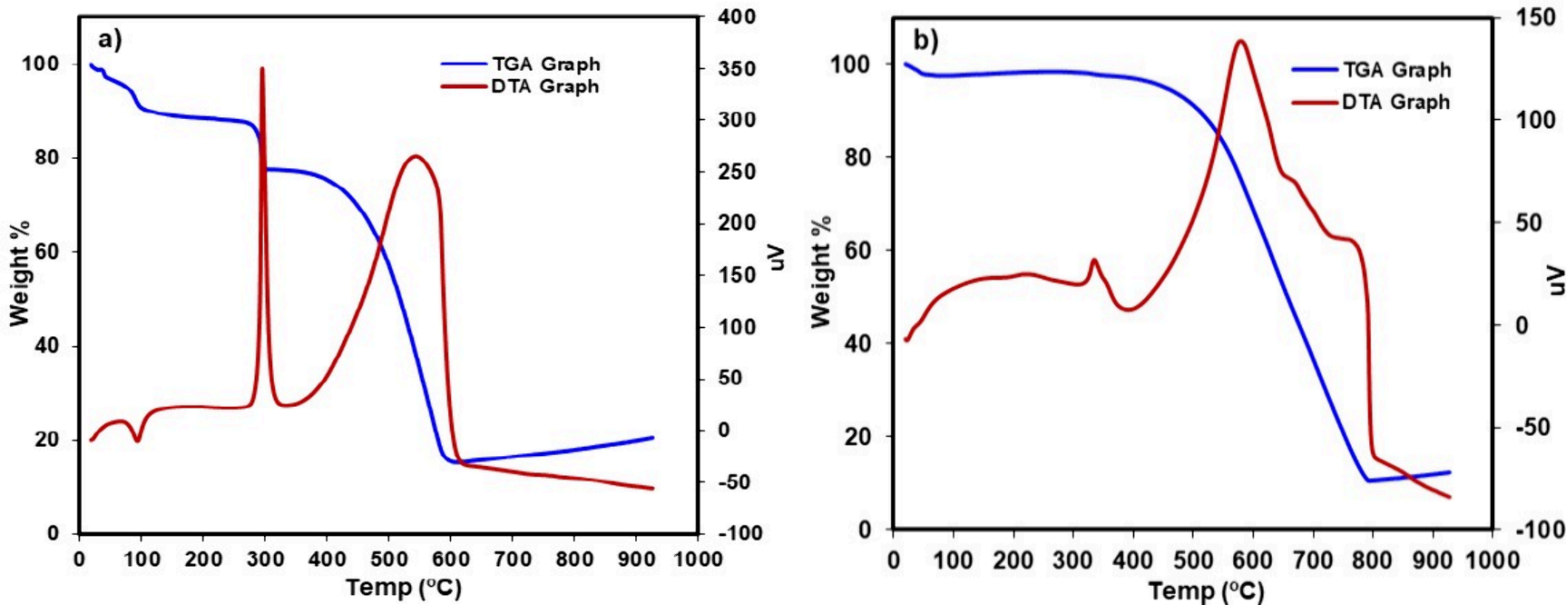
TGA and DTA graphs of Ni–Pd/MWCNTs (a) before the flow reaction and (b) after the flow reaction.

Thermogravimetric analysis (TGA) and differential thermal analysis (DTA) of the Ni–Pd/MWNCTs before and after the flow reaction were also investigated (Figure 8). As shown in Figure 8a for the catalyst before the flow reaction, the first degradation occurred at 73 °C with a small weight loss of 5–7% due to the loss of the physiosorbed and chemisorbed water molecules in the sample. The second degradation step was observed at the temperature of 298 °C with a weight loss of 15–20%, and the third degradation occurred at 370–600 °C, where a weight loss of 30–80% was steadily noticed most likely as a result of MWCNTs degradation to carbon residues along with Ni–Pd nanoparticles. The major degradation for Ni–Pd/MWCNTs after the flow reaction was observed at 480–775 °C with a weight loss of 10–80% (Figure 8b).

FTIR of the corresponding Ni–Pd/MWCNTs before and after the flow reaction demonstrated the characteristic peaks of MWCNTs with the stretching vibration at 3400 cm^–1^ due to the OH group on multiwalled carbon as well as two noticeable vibrations at 1587 and 1450 cm^–1^, most likely due to the C=C double bond and the C–O single bond, respectively, distributed on the surface of CNTs. (Figure S5).

### Diversity of Substrates in Flow Reaction

To generalize the above methodology, the versatility of the continuous flow process for the preparation of other biphenyl products utilizing a broad range of functional groups was investigated. For this purpose, the Ni–Pd/MWCNTs (Ni, 7.9%; Pd, 0.81%) nanoparticles were used due to the superior catalytic activity and greater throughput over the longer continuous runtime. As illustrated in Table 3, the Suzuki coupling of the variously substituted aryl iodide and functionalized boronic acid reagents was carried out using 300 mg of Ni–Pd/MWCNTs nanoparticles packed in a glass column. The reagents were mixed in H_2_O:EtOH:1,4-dioxane (1:1:1) in the presence of potassium carbonate as the base and pumped through the packed column at the specific flow rate of 0.2 mL/min at 130 °C. In this regard, aryl iodide including both electron donor and withdrawing groups was successfully incorporated into the coupling products. For instance, with the nitro group (**1b**) a quantitative steady-state yield was observed with the residence time (*t*_R_) of 2 min. Both aldehyde (**1c**) and nitrile (**1e**) can also be incorporated into the product in high yields with short residence time. The electron donor substituent such as isopropyl (**1d**) affording a moderate steady-state yield of 92% and *t*_R_ of 2.5 min. Nitrogen containing heterocyclic substrates such as pyrazine (**1f**), pyridine (**1h**), and pyrimidine (**1i**) are excellent coupling partners, providing a high yield of corresponding Suzuki products in flow. The method allows for steady-state continuous preparation of these important biologically active core structures utilizing our solid-packed catalyst. Notably, the corresponding aryl bromide could also be effectively employed as coupling partners providing high steady-state yields of the Suzuki products (**1b**, **1d**, **1e**, and **1f**) in continuous flow. Remarkably, we found that the same column of the catalyst could be used for multiple diversity reactions in flow without any noticeable loss of catalytic activity (**1c** and **1h**).

**Table 3:**
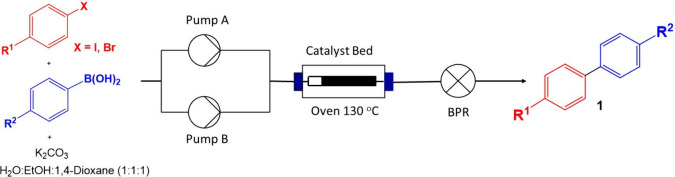

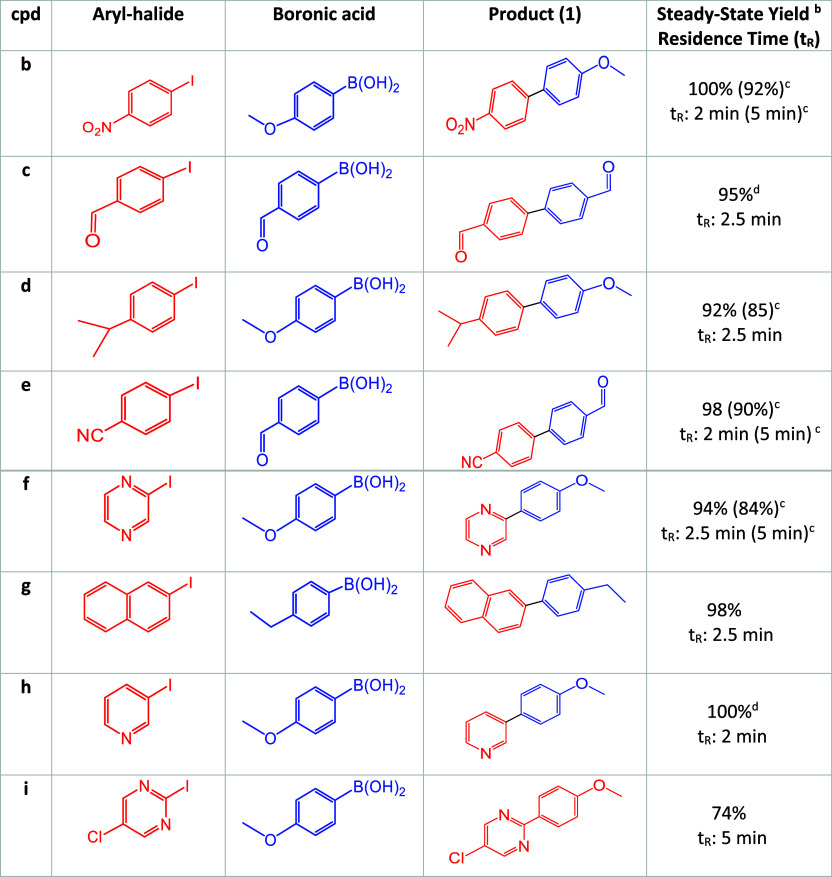
Diversity of the Suzuki coupling reactions in continuous flow using Ni–Pd/MWCNTs nanoparticles.^a^

a Reaction conditions: aryl halides (1.3 mmol, 1 equiv), aryl boronic acids (1.56 mmol, 1.2 equiv), and potassium carbonate (3.9 mmol, 3 equiv) in a mixture of H_2_O:EtOH:1,4-dioxane (300 mL) were passed through the reactor column (Omnifit, 15 mm o.d., 10 cm length) packed with Ni–Pd/MWCNTs nanoparticles (300 mg) at 0.2 mL/min while maintaining a reaction temperature of 130 °C using a hotcoil reactor.

b The steady-state yield (SSY) % was determined by GC-MS.

c Yields and residence time (*t*_R_) in parentheses correspond to aryl bromide.

d The same column of catalyst was used for these reactions.

The efficiency of this immobilized catalytic system toward the Suzuki cross-coupling reaction of functionalized substrates was further investigated by measuring the significant performance criteria such as the product throughput and space–time yield for each of the diversity reactions in Table 3. For this purpose, a solution of aryl halides (1.3 mmol), the corresponding boronic acid (1.56 mmol), and potassium carbonate (3.9 mmol) in a mixture of 300 mL of H_2_O:EtOH:1,4-dioxane (1:1:1) were pumped through the packed glass column containing Ni–Pd/MWCNTs nanoparticles (300 mg) at the specific flow rate indicated in Table 4. The temperature of the flow system was maintained at 130 °C by using a hotcoil reactor. The catalytic activity was measured by determining the minimum residence time required to achieve a minimum product threshold conversion of 90% and above. Consumption of starting materials and product formation were continuously monitored by GC-MS spectroscopy. As shown in Table 4, for aryl iodides **1b**–**1h** an optimal flow rate of 0.4–0.5 mL/min is required to obtain a high steady-state yield of 90% and above during the entire run time (10–13 h). Under these conditions, a product throughput of 0.1–0.13 mmol h^–1^ and the space–time yield of 94–130 mmol L^–1^ h^–1^ were accomplished. Notably, the reactions are performed in a short residence time of 2–2.5 min.

**Table 4:** Measuring performance criteria for Suzuki cross-coupling reactions in continuous flow^a^

Products	Flow Rate (mL/min)^c^	Run Time (h)	SSY (%)^b^	*t*_R_^c^ (min)	Throughput^c^ (mmol h^–1^)	STY^c^^,^^d^ (mmol L^–1^ h^–1^)
**1b**	0.5 (0.2)	10 (25)	100 (92)	2 (5)	0.13 (0.05)	130 (48)
**1c**	0.4	12.5	95	2.5	0.10	99
**1d**	0.4 (0.2)	12 (25)	92 (85)	2.5 (5)	0.1 (0.04)	96 (44)
**1e**	0.5 (0.2)	10 (25)	98 (90)	2 (5)	0.13 (0.05)	127 (47)
**1f**	0.4 (0.2)	12.5 (25)	94 (90)	2.5 (5)	0.10 (0.04)	98 (44)
**1g**	0.4	12.5	90	2.5	0.09	94
**1h**	0.5	10	100	2	0.13	130
**1i**	0.2	25	74	5	0.04	39

a Reaction conditions: aryl halides (1.3 mmol, 1 equiv), aryl boronic acids (1.56 mmol, 1.2 equiv), potassium carbonate (3.9 mmol, 3 equiv) in a mixture of H_2_O:EtOH:1,4-dioxane (300 mL) were passed through the reactor column (Omnifit, 15 mm o.d., 10 cm length) packed with Ni–Pd/MWCNTs nanoparticles (300 mg) at the indicated flow rates while maintaining a reaction temperature of 130 °C using a hotcoil reactor.

b The steady-state yield % was determined by GC-MS.

c Data in the parentheses correspond to aryl bromide.

d Space–time yield (STY) was measured based on the volume of the solid catalyst in the glass column.

On the contrary, 2-iodo-5-chloropyrimidine (**1i**) required a slower flow rate of 0.2 mL/min to afford a steady-state yield of 74% with a product throughput of 0.04 mmol h^–1^ and space–time yield of 39 mmol L^–1^ h^–1^ over a longer run time of 25 h. Similarly, the aryl bromides also required a slower flow rate of 0.2 mL/min to achieve a high steady-state yield of above 85% and above (**1b**, **1d**, **1e**, and **1f**). The maximum throughput for substituted aryl bromides was 0.05 mmol h^–1^ affording a space–time yield of 44–48 mmol L^–1^ h^–1^, while an optimal residence time of 5 min was required. The overall goal of this effort was to develop an efficient continuous process methodology that would complement and leverage the reactivity observed under batch reaction conditions (Table 4).

## Conclusions

In summary, bimetallic nanoparticles containing nickel and palladium (Ni, 7.9%; Pd, 0.81%) supported on multiwalled carbon nanotubes (Ni–Pd/MWCNTs) were successfully employed as a remarkable catalyst for Suzuki cross-coupling reactions in continuous flow using a packed bed column. The catalyst in the column was prepared by simple mixing of the precursor nickel and palladium salts with multiwalled carbon nanotubes followed by mechanical shaking in a ball-mill at ambient temperature. This rapid, straightforward, and inexpensive method with no necessity for any solvent or reducing agent provided access to the bulk quantity of the desired supported nanoparticles at once, making it ideal for scaling up the catalytic process in flow. Considering the potential viability of this catalyst, the concentration of palladium in this bimetallic system can be lowered to 0.81% according to ICP-OES results and replaced by more available and cheaper nickel, thus still achieving a great catalytic activity and performance. Moreover, small particle sizes of 1–5 nm (Pd nanoparticles) with uniform distribution of the particles on the surface of the carbon nanotubes played an important role for the greater reactivity. The as-prepared Ni–Pd/MWCNTs nanoparticles exhibited superior catalytic activity for Suzuki reactions in flow utilizing a wide array of functionalized aryl halides and phenylboronic acid affording great steady-state yields with high throughput in a short residence time (*t*_R_) in each reaction. The rapid, convenient, and scalable access to the bulk amount of catalyst makes this effort an ideal approach to develop a continuous flow technology with direct pharmaceutical and industrial applications for the synthesis of major carbon–carbon bond formation through the Suzuki cross-coupling reaction. Studies toward the potential use of this method for other cross-coupling reactions under continuous flow conditions are ongoing in our laboratory.

## Supporting Information Available

Additional characterization data and ^1^H/^13^C NMR spectra of all products (PDF)
