# Supplementary material for: Bimetallic Nickel–Palladium Nanoparticles Supported on Multiwalled Carbon Nanotubes for Suzuki Cross-Coupling Reactions in Continuous Flow
Source: Ind Eng Chem Res. 2025 Jan 7;64(3):1427–38. doi: 10.1021/acs.iecr.4c02922 (PMC11760624; doi:10.1021/acs.iecr.4c02922)

Bimetallic Nickel-Palladium Nanoparticles Supported on Multiwalled Carbon Nanotubes for Suzuki Cross-Coupling Reactions in Continuous Flow

Katherine A. Wilson, Harlee B. Winkleman, and Ali R. Siamaki*

Department of Chemistry, Physics, and Materials Science, Fayetteville State University, Fayetteville, NC, USA 28301

Supporting Information

Experimental Procedures--**S 2**

Additional Characterization Data--- **S 4**

^{1}H and ^{13}C NMR Data--**S 7**

^{1}H and ^{13}C NMR Spectra of Compounds in Table 3-----------------------------**S 10**

General Methods

Multiwalled carbon nanotubes (MWCNTs) of 30–50 nm were purchased from ACS Material, Advanced Chemicals Supplier. Nickel(II) acetate tetrahydrate and palladium(II) acetate were purchased from Sigma-Aldrich. Aryl iodides and bromides, phenylboronic acid, and other substituted boronic acids were obtained from Sigma-Aldrich, Alfa Aesar, and ACROS Organics and used as received. A mixture of deionized water, ethanol, and 1,4-dioxane (1:1:1) was used to dissolve the reagents and pump them through the flow reactor. Shimadzu GC-MS QP2010 SE was used to complete the gas chromatography–mass spectroscopy (GC-MS) analysis of the organic products. Thermo Fisher Talos F200X G2, a 200 kV FEG (field emission gun) at Analytical Instrumentation Facilities at North Carolina State University (AIF) was employed to perform the TEM analysis of the catalysts. Nuclear magnetic resonance spectroscopy measurements, including those of ^{1}H and ^{13}C NMR, were obtained utilizing a JEOL 400 MHz spectrometer. A Uniqsis FlowLab reactor equipped with high-pressure pumps, a hotcoil, and a glass column of 10 mm i.d., (15 mm o.d.) PTFE/PEEK with tubing, being adjustable, was used for continuous flow reactions. A Kratos Axis Supra X-ray photoelectron spectrometer at University of North Carolina—Chapel Hill (UNC—Chapel Hill) instrumentation facilities (CHANL) was used to complete the XPS analysis. Inductively coupled plasma-optical emission spectroscopy (ICP-OES) analysis was completed using iCAP 7200 (Thermo Fisher Scientific). A Rigaku MiniFlex 600 X-ray diffractometer (XRD) was used to conduct the XRD analysis of the solid catalysts before and after the flow reaction. Thermal gravimetric analyzer Shimadzu DTG-60A was employed for TGA/DTA analysis. An Anton Paar high-vacuum physisorption analyzer Autosorb iQ was used for the BET isotherm surface area and pore size measurements. A Thermo Fisher Scientific Nicolet iS50 FTIR spectrometer was used for FTIR analysis of the samples.

Synthesis of Solid Supported Catalysts

The following compositions were prepared and screened to investigate the best catalytic system for Suzuki reactions in flow. The previously published procedure was used with slight modification in concentration of the metals to prepare the catalysts. Each sample was placed in zirconium ceramic vials and subjected to extensive mechanical shaking for 45 min at ambient temperature using a SPEX 8000 M ball-mill mixer. The mechanical movement of the ball-mill provided 1060 cycles per minute with 5.9 cm back and forth and 2.5 cm side to side. The following concentrations were prepared:

Ni–Pd/MWCNTs (10% Ni, 1% Pd): Nickel acetate tetrahydrate (42.39 mg, 10% Ni content) and palladium acetate (2.11 mg, 1% Pd content) were mixed with multiwalled carbon nanotubes (MWCNTs) (89 mg).

Ni/MWCNTs: (20% Ni): Nickel acetate tetrahydrate (84.78 mg, 20% Ni content) was mixed with MWCNTs (80 mg).

Ni/MWCNTs: (10% Ni): Nickel acetate tetrahydrate (42.39 mg, 10% Ni content) was added to MWCNTs (80 mg).

Pd/MWCNTs: (1% Pd): Palladium acetate (2.11mg, 1% Pd content) was added to MWCNTs (99 mg).

General procedure for Continuous flow Suzuki cross-coupling reactions

The continuous reaction was performed using a Uniqsis FlowLab reactor equipped with two high-pressure pumps, a hotcoil oven, and an Omnifit glass column in which the solid supported catalyst can be introduced and packed. A solution of 4-iodobenzaldehyde (300 mg, 1.29 mmol), phenylboronic acid (188.7 mg, 1.55 mmol), and potassium carbonate (534 mg, 3.87 mmol) in a mixture of H_2O:EtOH:1,4-dioxane (150 mL) was pumped through the reactor column (Omnifit, 15 mm o.d., 10 cm length) packed with 300 mg of the corresponding catalyst at a specific flow rate while maintaining a reaction temperature of 130 °C using a hotcoil oven. The flow rate was adjusted accordingly to maintain a high steady-state yield. The product was collected in fractions (5–10 mL), and each fraction was analyzed by GC-MS spectroscopy. For prolonged on-stream reaction in Figure 3, 4-iodobenzaldehyde (2.4 g, 10.3 mmol), phenylboronic acid (1.5 g, 12.4 mmol), and potassium carbonate (4.3 g, 31.02 mmol) in a mixture of H_2O:EtOH:1,4-dioxane (1.2 L) were passed through the reactor column (Omnifit, 15 mm o.d., 10 cm length) packed with 300 mg of the corresponding catalyst at a specific flow rate while maintaining a reaction temperature of 130 °C using an a hotcoil reactor. The reaction mixtures were collected in fractions and analyzed by GC-MS spectroscopy. The fractions were combined and extracted with dichloromethane (2 × 5 mL). The organic layers were decanted, dried over sodium sulfate, and filtered. After removal of the solvent under reduced pressure, the final product was subjected to flash chromatography using hexane:ethyl acetate.

Procedure for continuous flow Suzuki cross-coupling reaction using functionalized substrates

A solution of aryl halides (1.3 mmol, 1 equiv), aryl boronic acids (1.56 mmol, 1.2 equiv), and potassium carbonate (3.9 mmol, 3 equiv) in a mixture of H_2O:EtOH:1,4-dioxane (300 mL) was pumped through the reactor column (Omnifit, 15 mm o.d., 10 cm length) packed with 300 mg of the corresponding catalyst at a specific flow rate while maintaining a reaction temperature of 130 °C using a hotcoil oven. The flow rate was adjusted accordingly to maintain a high steady-state yield. The product was collected in fractions (5–10 mL), and each fraction was analyzed using GC-MS technique. For the recovery of the final products, all of the fractions were combined and extracted using dichloromethane (2 × 5 mL). The organic layers were decanted, dried over sodium sulfate, and filtered. The final product was purified by flash chromatography on silica gel using hexane:ethyl acetate as the solvent.

Additional Characterization Data:

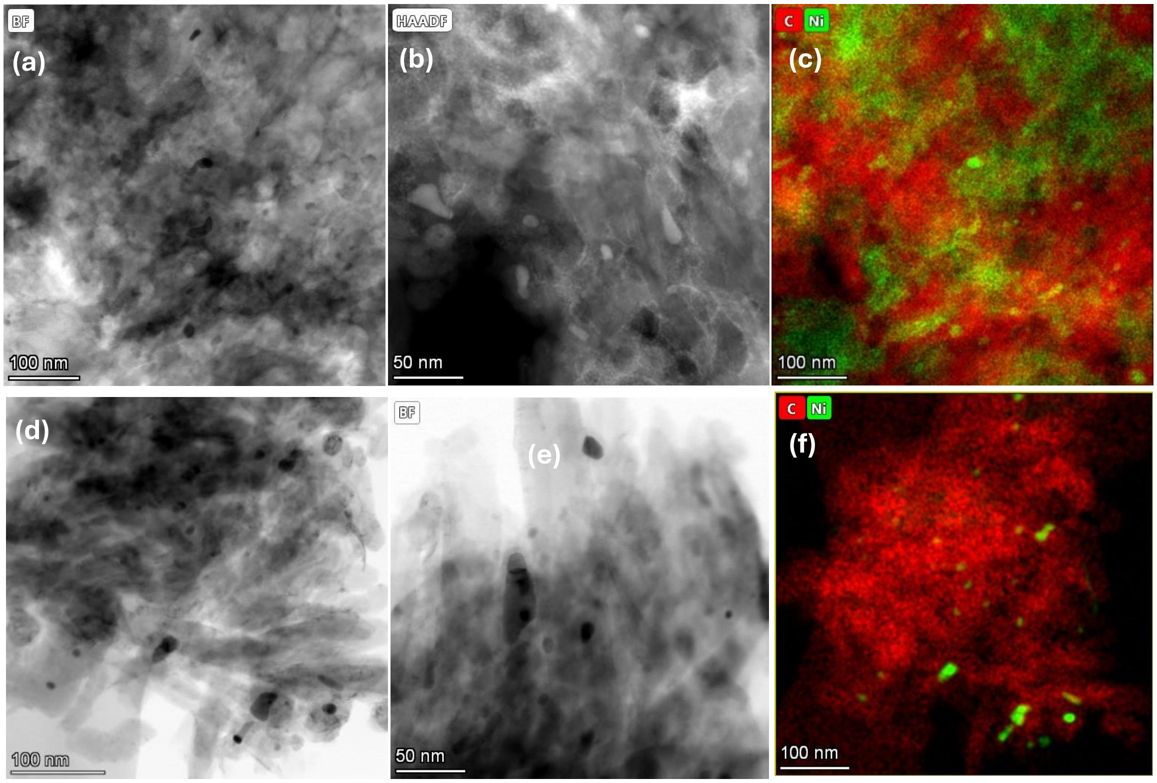

Figure S1: TEM images and EDS mapping of S1(a-c)) Ni/MWCNTs (Ni:18.5%) before the reaction and S1(d-f) after the reaction.

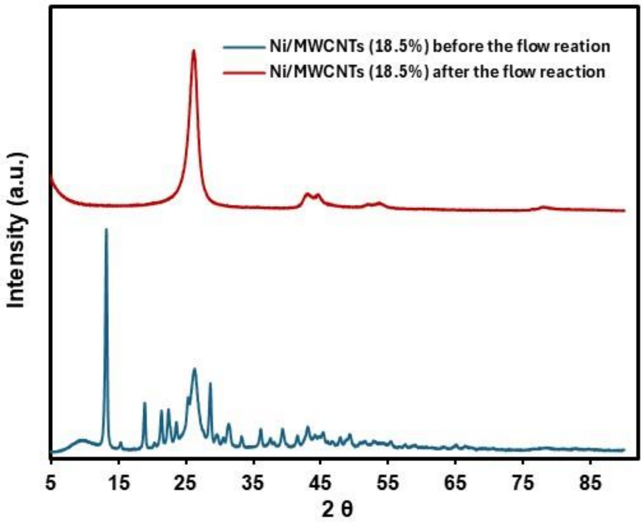

Figure S2. XRD pattern of Ni/MWCNTs (18.5%) before and after the flow reaction.

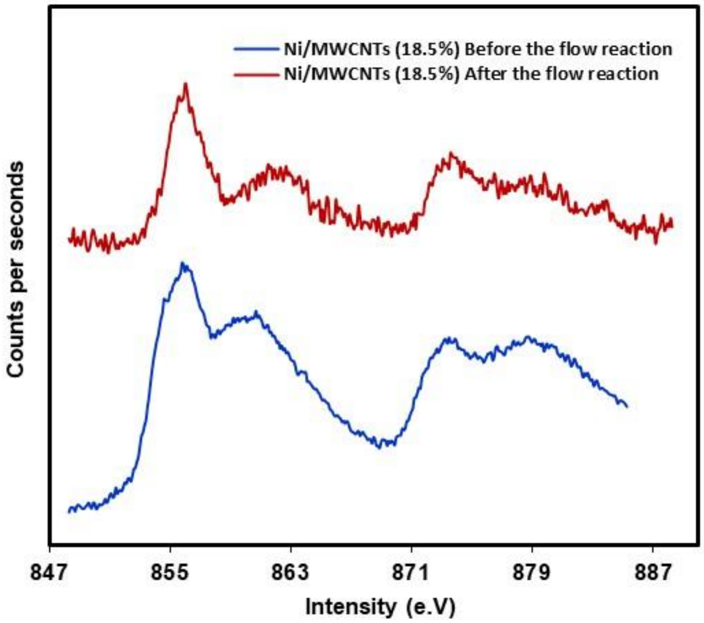

Figure S3. XPS spectra of Ni/MWCNTs (18.5%) before and after the flow reaction.

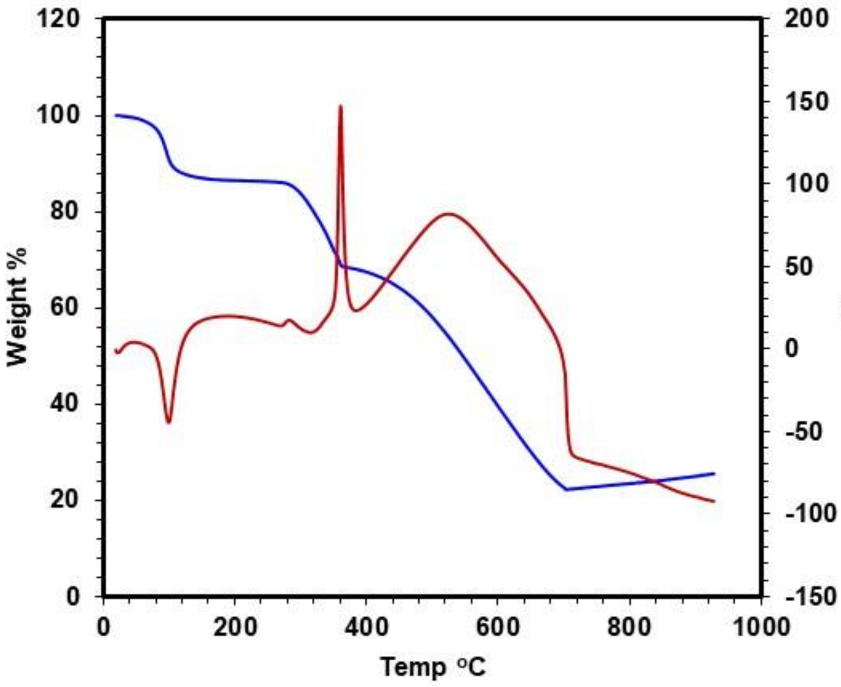

Figure S4. TGA/TDA spectra of Ni/MWCNTs (18.5%) before and after the flow reaction.

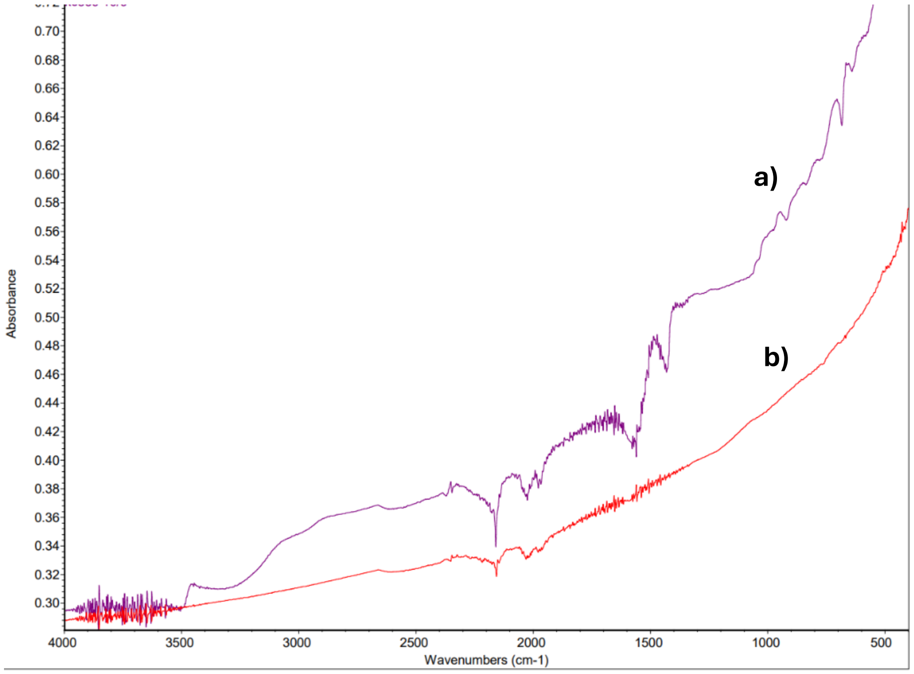

Figure S5. FTIR Spectra of Ni-Pd/MWCNTs (Ni:7.9%, Pd:0.81%) a) before the flow reaction b) after the flow reaction.

Spectroscopic Data for Table 3:

1a: [1,1'-biphenyl]-4-carbaldehyde

^{1}H-NMR (400 MHz, CHLOROFORM-D) δ 10.04 (s, 1H), 7.94 (d, J = 8.2 Hz, 2H), 7.74 (d, J = 8.0 Hz, 2H), 7.63 (d, J = 7.3 Hz, 2H), 7.47 (t, J = 7.4 Hz, 2H), 7.42 (d, J = 7.1 Hz, 1H).

^{13}C-NMR (101 MHz, CHLOROFORM-D) δ 192.12, 147.30, 139.80, 135.25, 130.40, 129.12, 128.59, 127.80, 127.47, 77.46, 77.35, 77.15, 76.83.

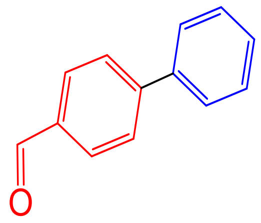

1b: 4-methoxy-4'-nitro-1,1'-biphenyl

^{1}H-NMR (400 MHz, CHLOROFORM-D) δ 8.25 (d, J = 8.9 Hz, 2H), 7.67 (d, J = 8.7 Hz, 2H), 7.57 (d, J = 8.7 Hz, 2H), 7.01 (d, J = 8.9 Hz, 2H), 3.86 (s, 3H).

^{13}C-NMR (101 MHz, CHLOROFORM-D) δ160.50, 147.29, 146.58, 131.13, 128.67, 127.15, 124.24, 114.68, 77.32, 77.12, 76.81, 55.52.

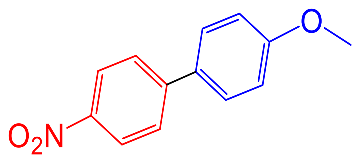

1c: [1,1'-biphenyl]-4,4'-dicarbaldehyde

^{1}H-NMR (400 MHz, CHLOROFORM-D) δ 10.07 (s, 2H), 7.98 (d, J = 8.0 Hz, 4H), 7.79 (d, J = 8.2 Hz, 4H).

^{13}C-NMR (101 MHz, CHLOROFORM-D) δ 191.88, 145.63, 136.03, 130.47, 128.13, 77.45, 77.34, 77.14, 76.82.

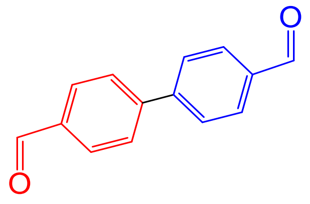

1d: 4-methoxy-4'-(propan-2-yl)-1,1'-biphenyl

^{1}H-NMR (400 MHz, CHLOROFORM-D) δ 7.49 (dd, J = 12.3, 8.5 Hz, 1H), 7.27 (d, J = 8.2 Hz, 0H), 6.95 (dd, J = 8.8, 2.2 Hz, 1H), 3.84 (s, 3H), 2.93 (s, 1H), 1.28 (d, J = 7.1 Hz, 6H).

^{13}C-NMR (101 MHz, CHLOROFORM-D) δ 158.96, 147.45, 138.44, 133.84, 128.10, 127.83, 126.90, 126.75, 114.20, 77.43, 77.32, 77.12, 76.80, 55.42, 33.85, 24.13.

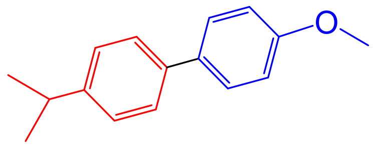

1e: 4'-formyl[1,1'-biphenyl]-4-carbonitrile

^{1}H-NMR (400 MHz, CHLOROFORM-D) δ 10.07 (s, 1H), 7.99 (d, J = 8.0 Hz, 2H), 7.75 (q, J = 8.9 Hz, 6H).

^{13}C-NMR (101 MHz, CHLOROFORM-D) δ 191.78, 145.00, 144.22, 136.17, 132.90, 130.54, 128.14, 128.03, 118.69, 112.22, 77.44, 77.33, 77.13, 76.80.

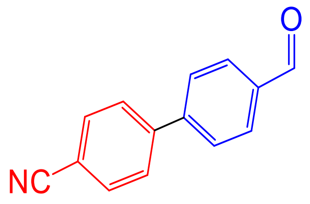

1f: 2-(4-methoxyphenyl)pyrazine

^{1}H-NMR (400 MHz, CHLOROFORM-D) δ 8.96 (s, 1H), 8.57 (s, 1H), 8.42 (d, J = 2.1 Hz, 1H), 7.96 (d, J = 8.7 Hz, 2H), 7.01 (d, J = 8.7 Hz, 2H), 3.86 (s, 3H).

^{13}C-NMR (101 MHz, CHLOROFORM-D) δ 161.25, 152.62, 144.11, 142.20, 141.73, 128.92, 128.38, 114.57, 77.44, 77.33, 77.13, 76.81, 55.50.

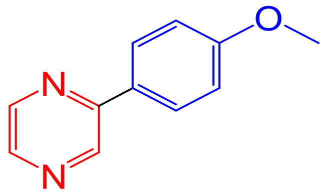

1g: 2-(4-ethylphenyl)naphthalene

^{1}H-NMR (400 MHz, ACETONE-D6) δ 8.13 (s, 1H), 7.94-7.96 (m, 2H), 7.89 (d, J = 7.5 Hz, 1H), 7.80 (d, J = 8.5 Hz, 1H), 7.69 (d, J = 8.0 Hz, 2H), 7.48 (t, J = 7.0 Hz, 2H), 7.33 (d, J = 8.0 Hz, 2H), 2.67 (d, J = 7.5 Hz, 2H), 1.23 (t, J = 7.7 Hz, 3H).

^{13}C-NMR (101 MHz, ACETONE-D6) δ 143.62, 138.33, 138.20, 133.99, 132.72, 128.50, 128.21, 127.61, 127.16, 126.33, 126.07, 125.90, 125.30, 125.18, 28.25, 15.29.

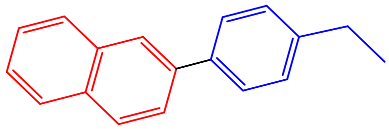

1h: 3-(4-methoxyphenyl)pyridine

^{1}H-NMR (400 MHz, CHLOROFORM-D) δ 8.79 (s, 1H), 8.51 (d, J = 4.1 Hz, 1H), 7.79 (d, J = 7.8 Hz, 1H), 7.49 (d, J = 8.5 Hz, 2H), 7.30 (s, 1H), 6.98 (d, J = 8.5 Hz, 2H), 3.82 (s, 3H).

^{13}C-NMR (101 MHz, CHLOROFORM-D) δ 159.81, 148.04, 147.93, 136.31, 133.95, 130.28, 128.30, 123.61, 114.61, 77.51, 77.39, 77.19, 76.88, 55.45.

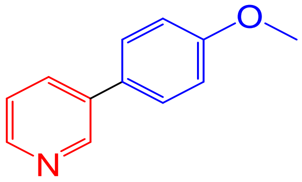

1i: 5-chloro-2-(4-methoxyphenyl)pyrimidine

^{1}H-NMR (400 MHz, CHLOROFORM-D) δ 8.77 (s, 2H), 7.48 (d, J = 8.7 Hz, 2H), 7.03 (d, J = 8.7 Hz, 2H), 3.85 (s, 3H).

^{13}C-NMR (101 MHz, CHLOROFORM-D) δ 160.75, 159.22, 157.06, 132.77, 128,20, 128.05, 125.18, 115.13, 77.44, 77.33, 77.13, 76.81, 55.56.

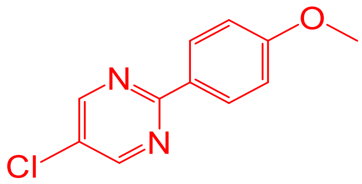

1a: [1,1'-biphenyl]-4-carbaldehyde ^{1}H NMR

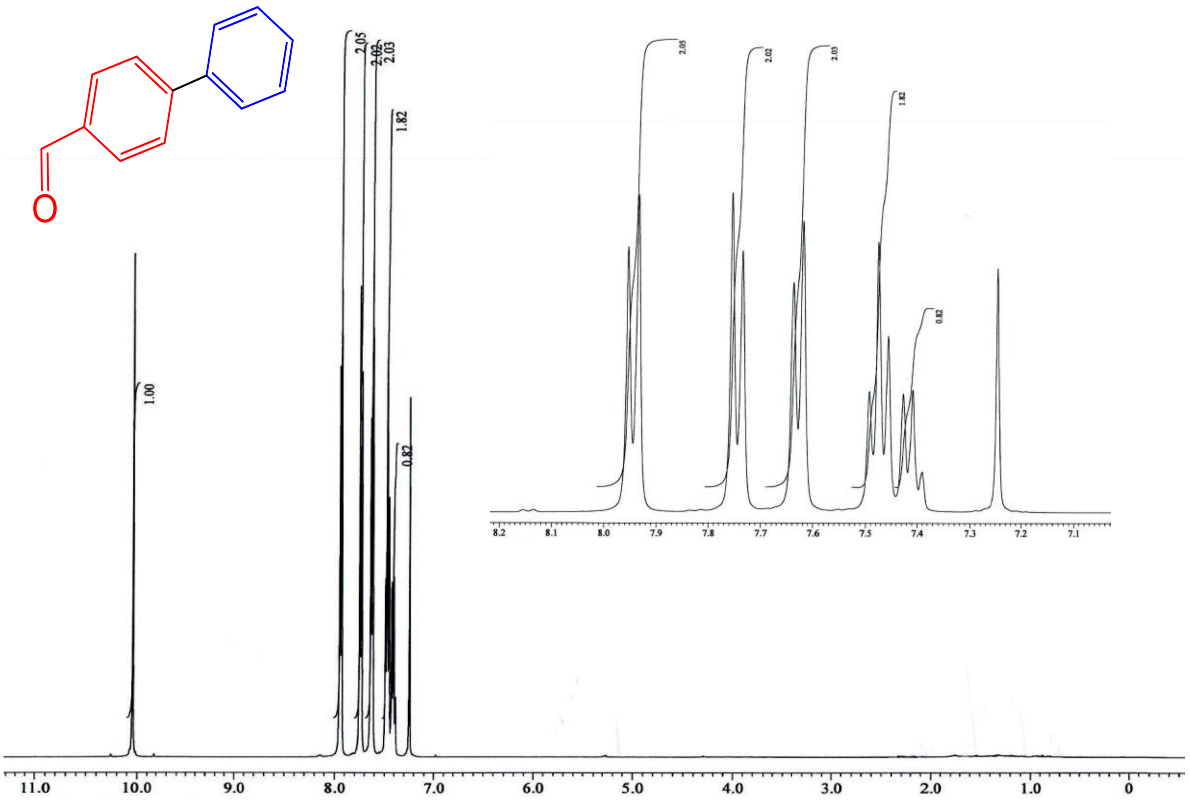

^{13}C NMR:

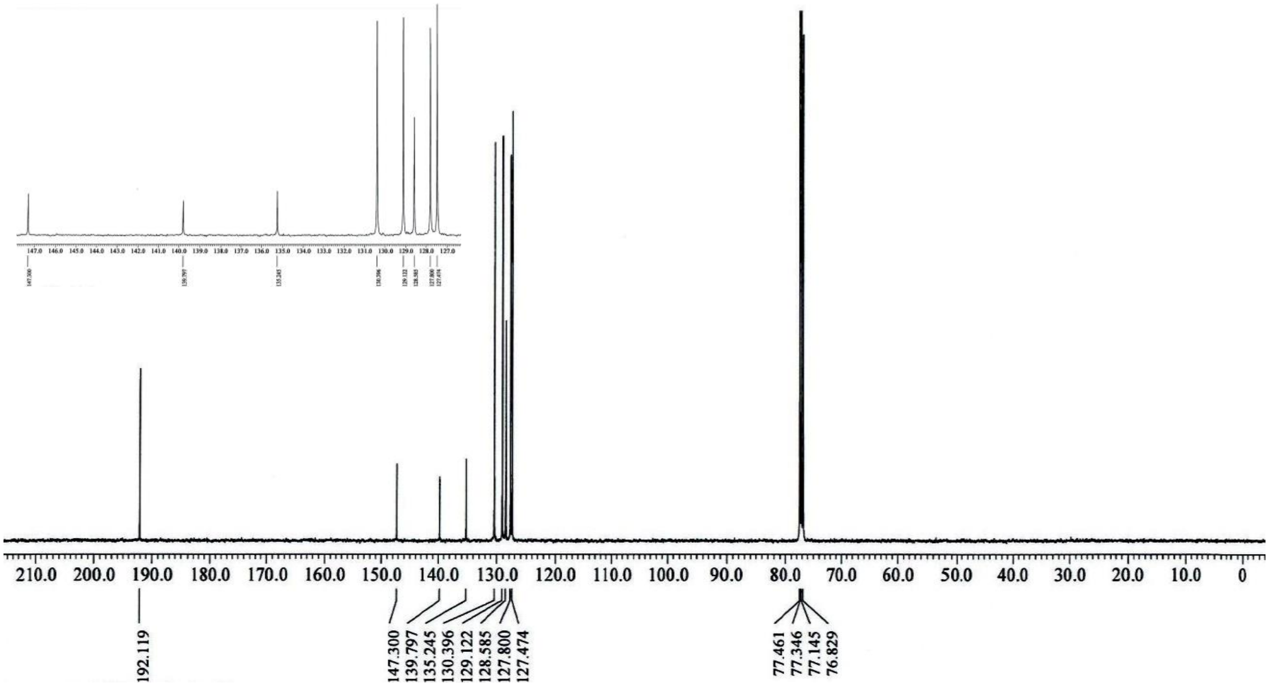

1b: 4-methoxy-4'-nitro-1,1'-biphenyl ^{1}H NMR

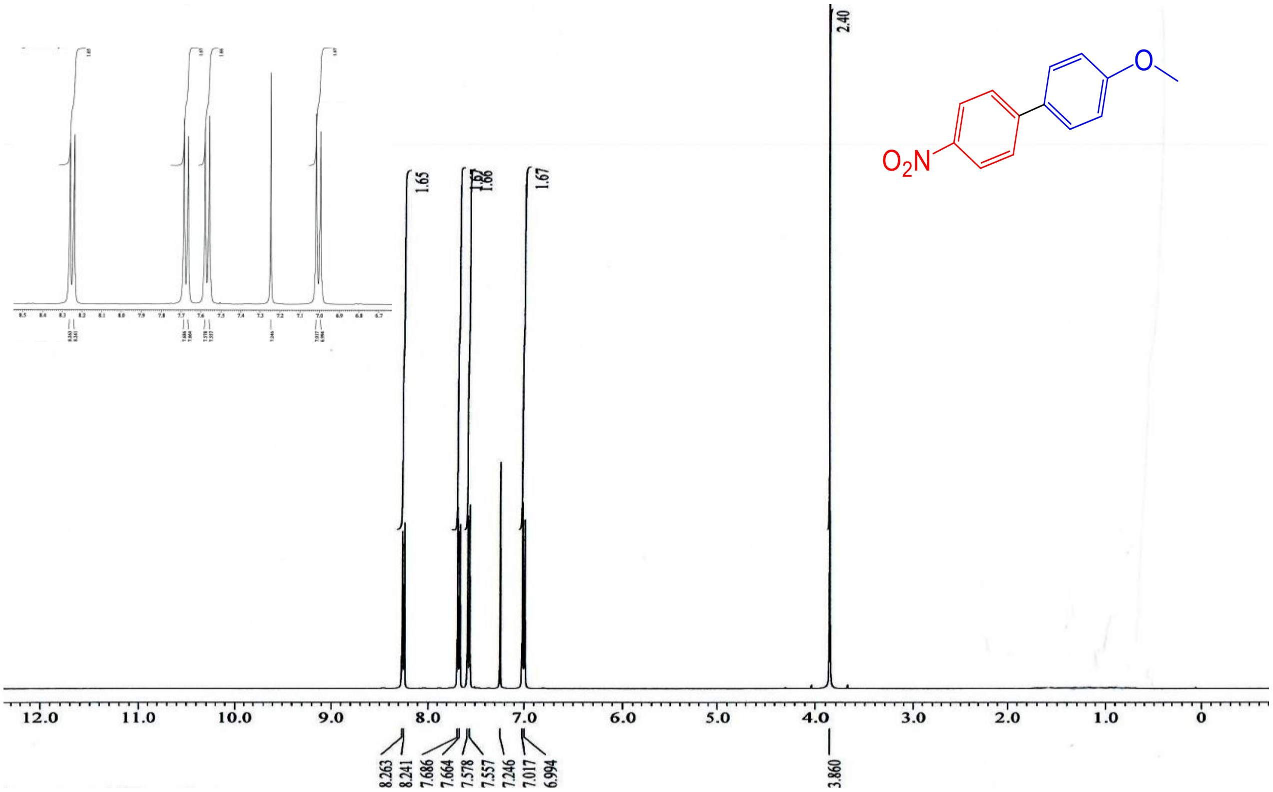

^{13}C NMR:

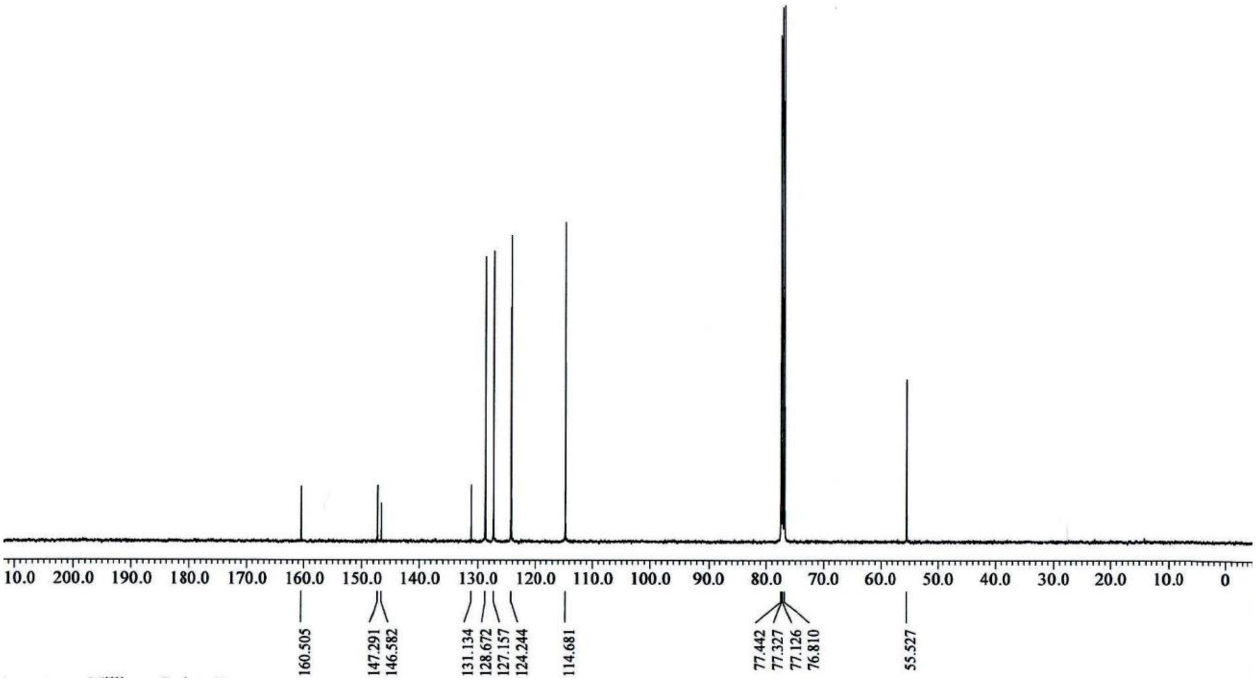

1c: [1,1'-biphenyl]-4,4'-dicarbaldehyde ^{1}H NMR

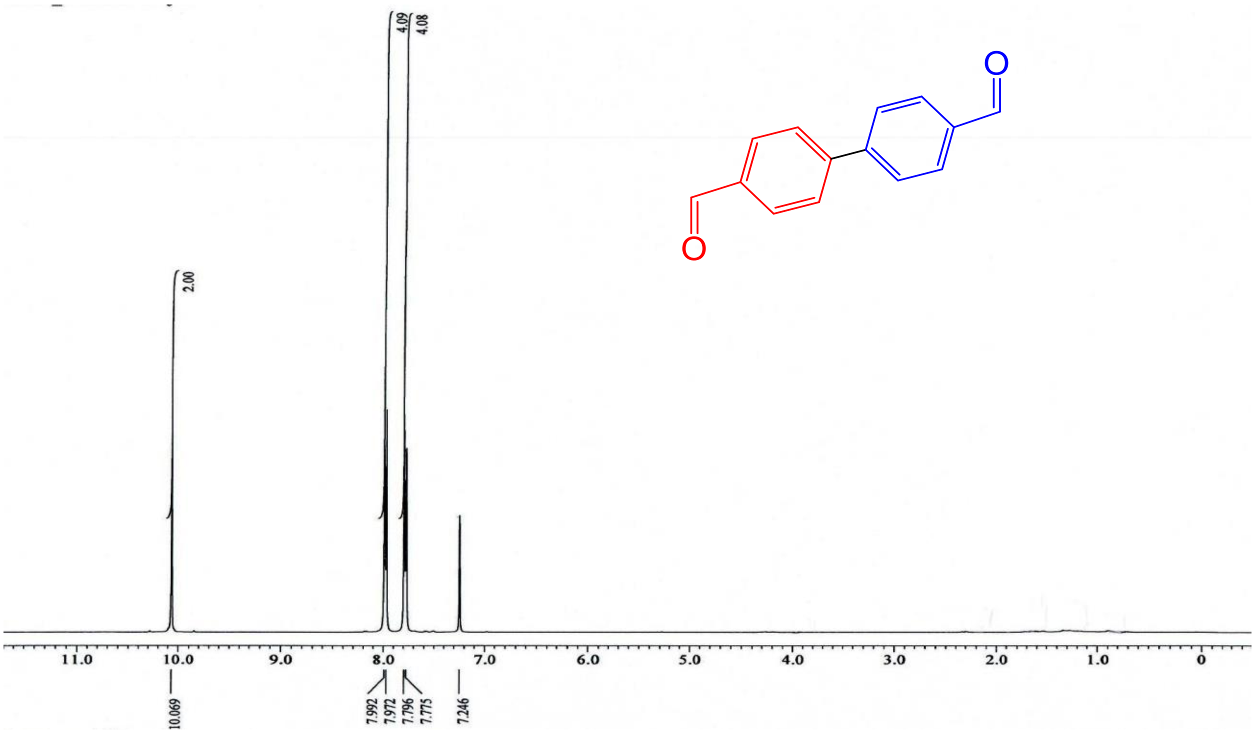

^{13}C NMR:

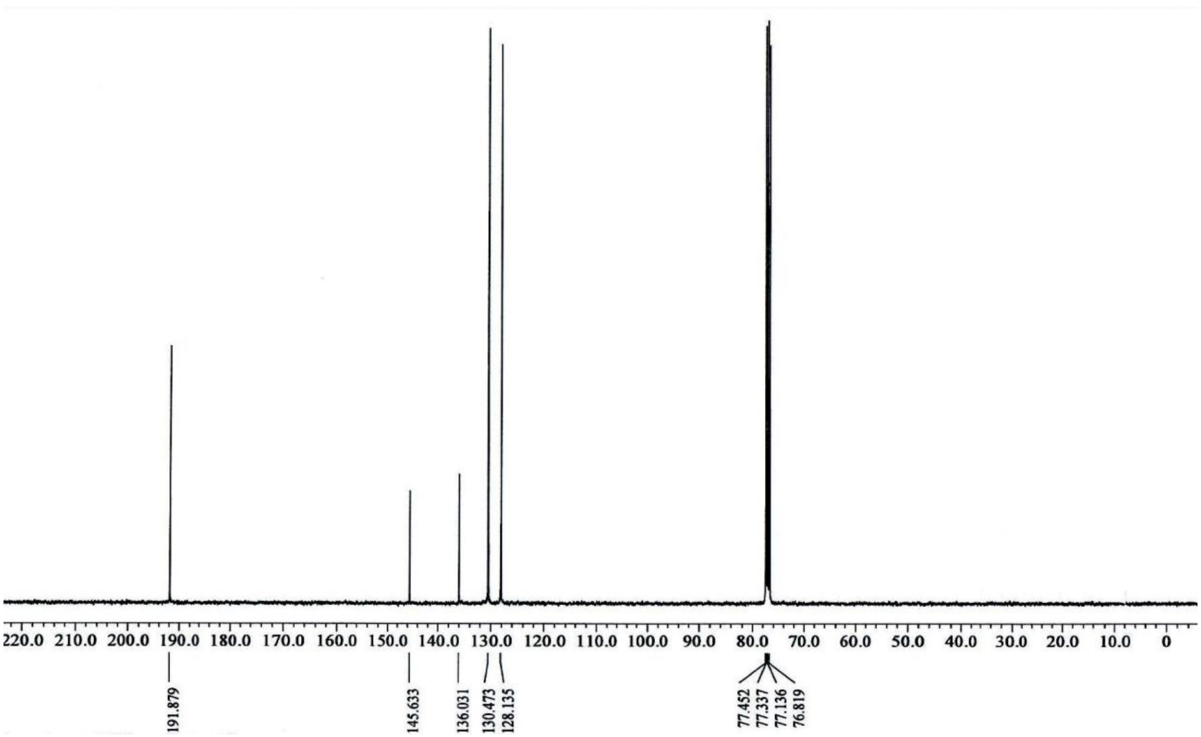

1d: 4-methoxy-4'-(propan-2-yl)-1,1'-biphenyl ^{1}H NMR

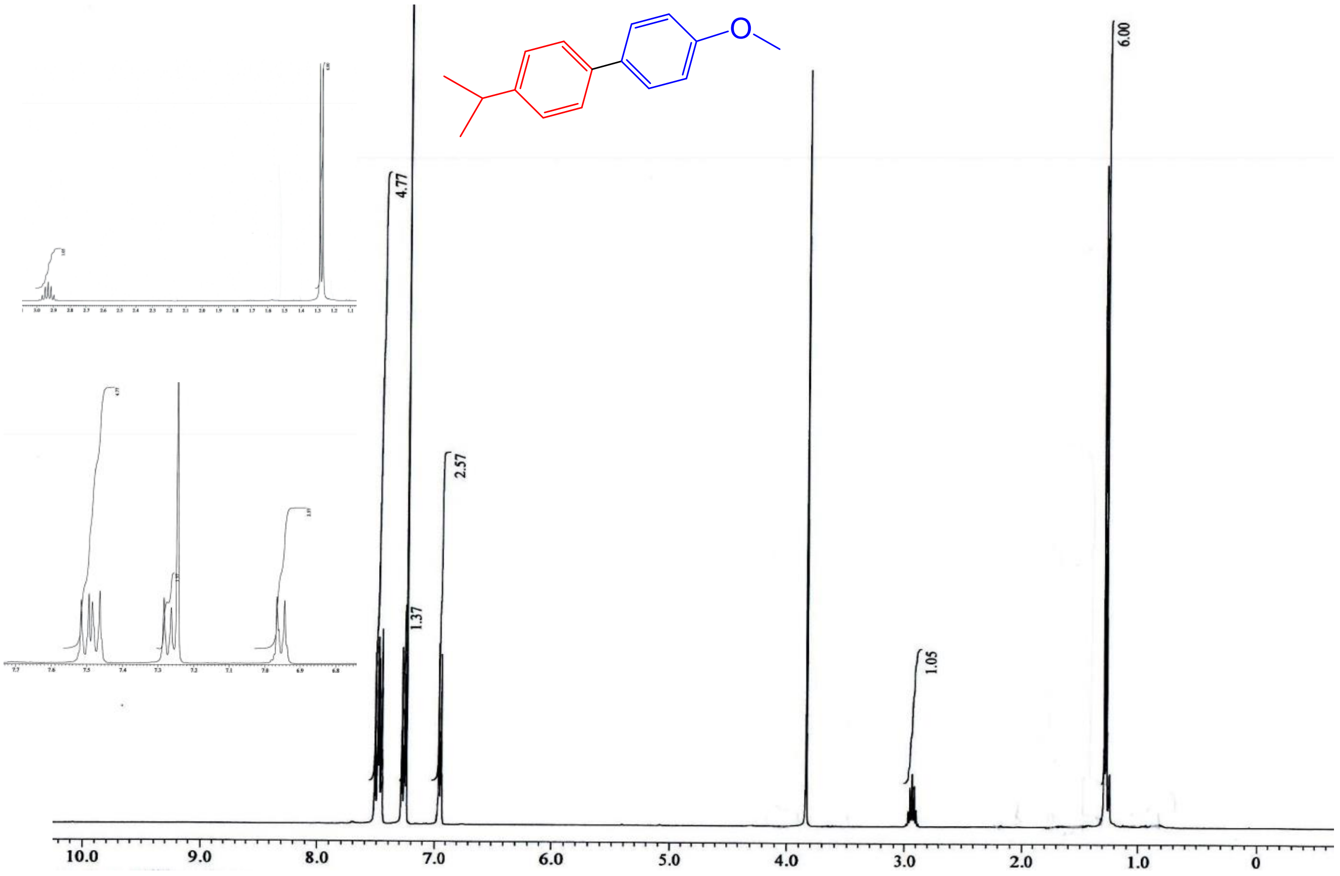

^{13}C NMR:

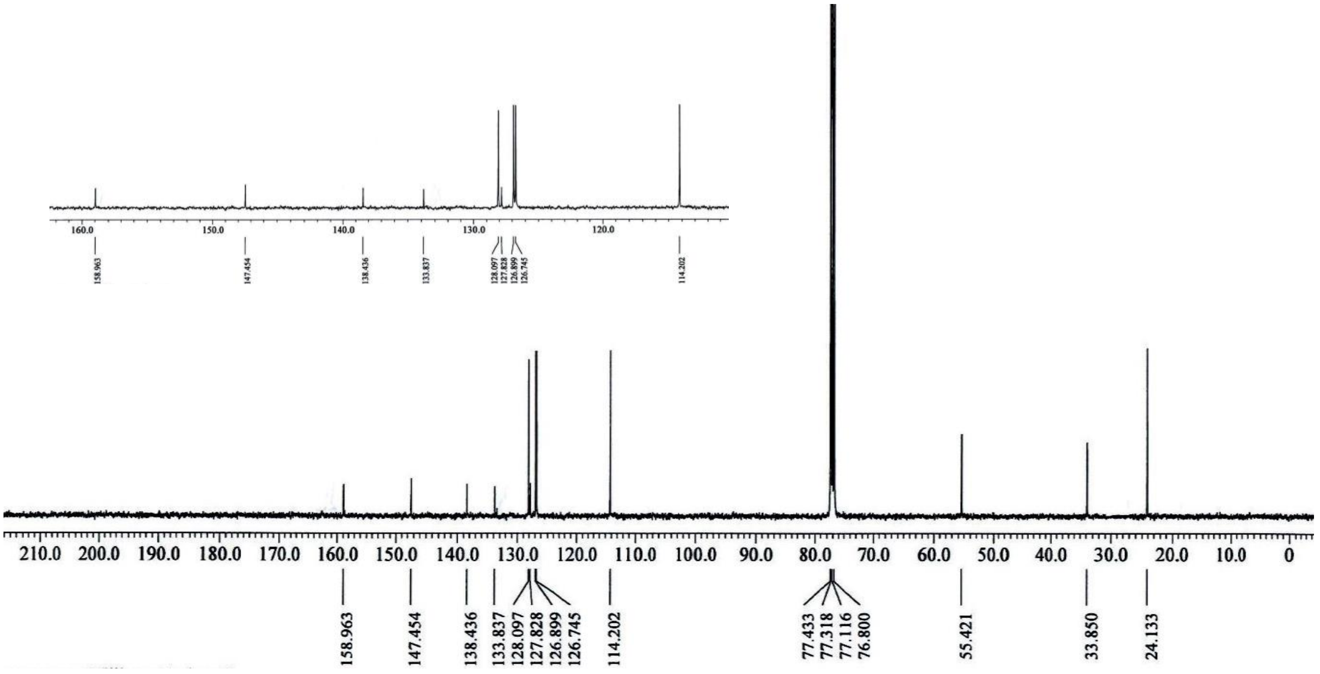

1e: 4'-formyl[1,1'-biphenyl]-4-carbonitrile ¹H NMR

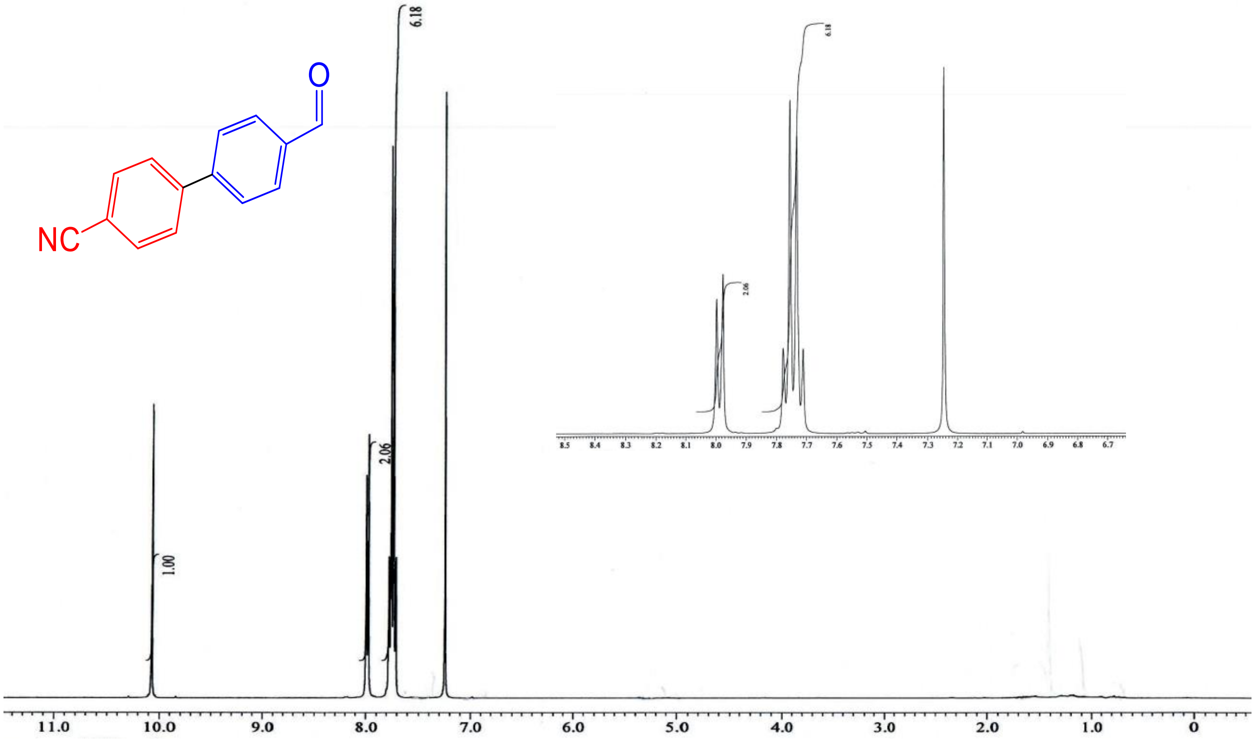

^{13}C NMR:

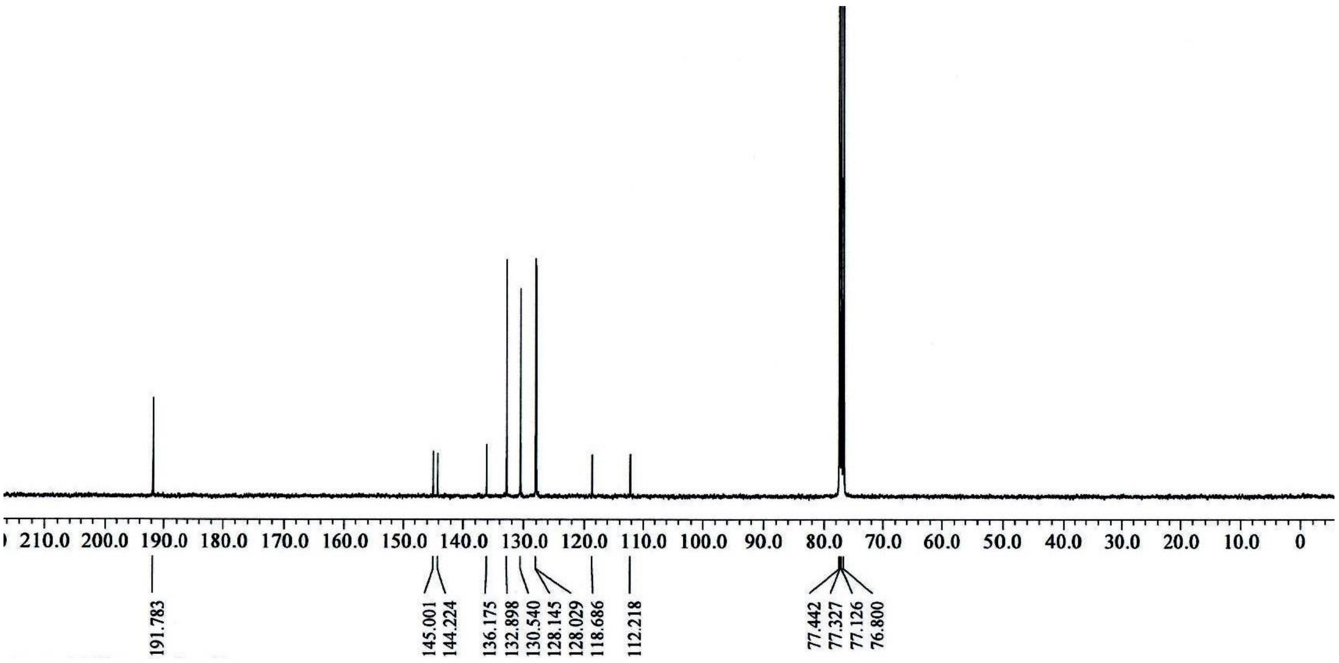

1f: 2-(4-methoxyphenyl)pyrazine ^{1}H NMR

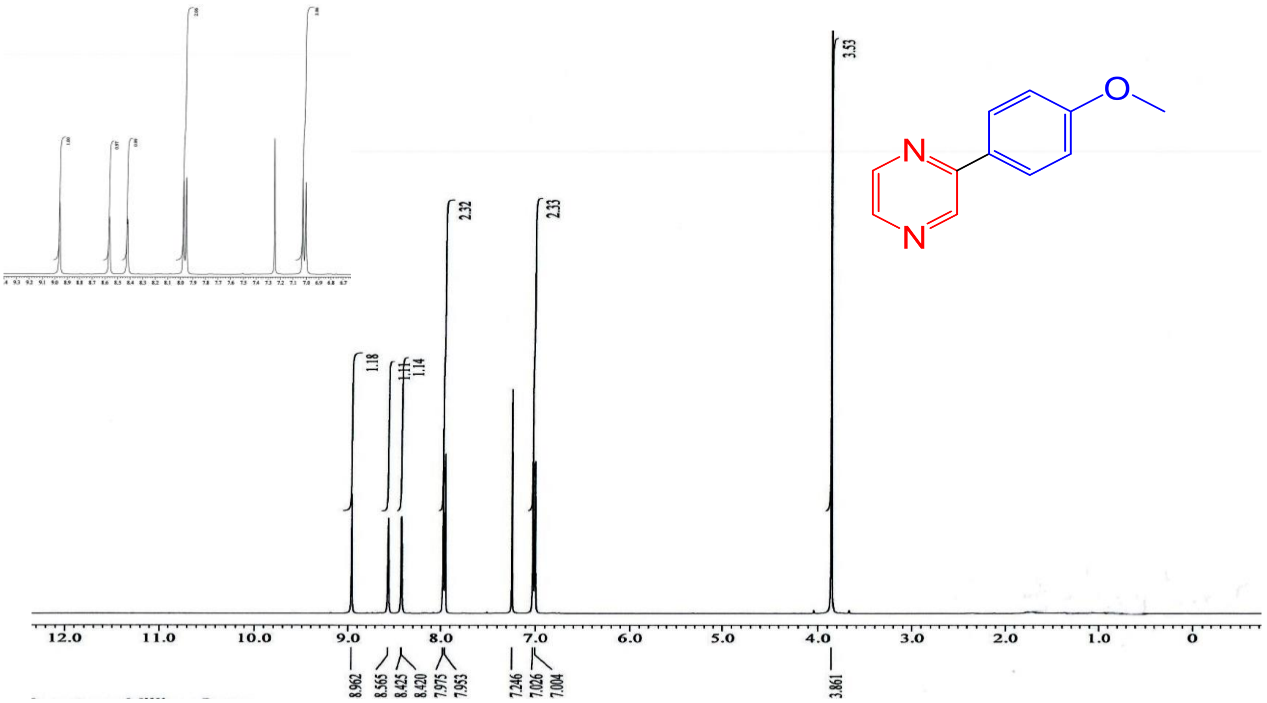

^{13}C NMR:

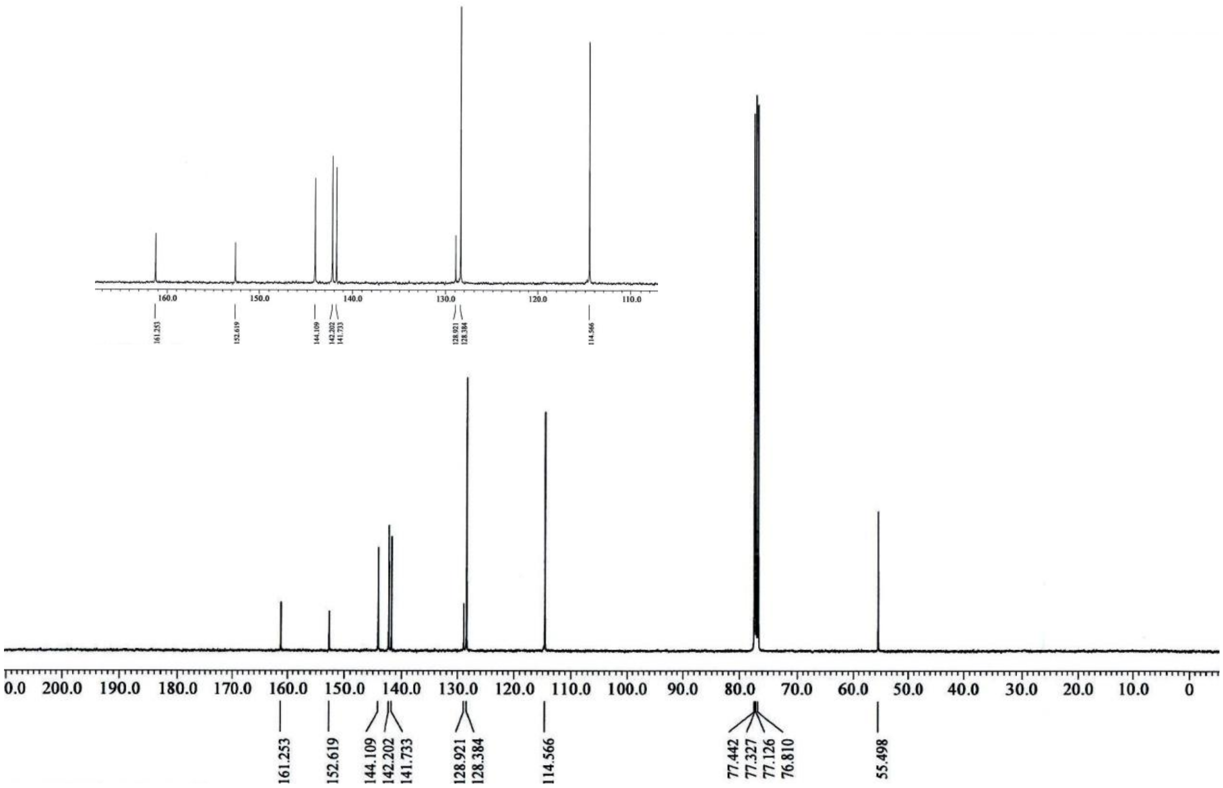

1g: 2-(4-ethylphenyl)naphthalene ^{1}H NMR

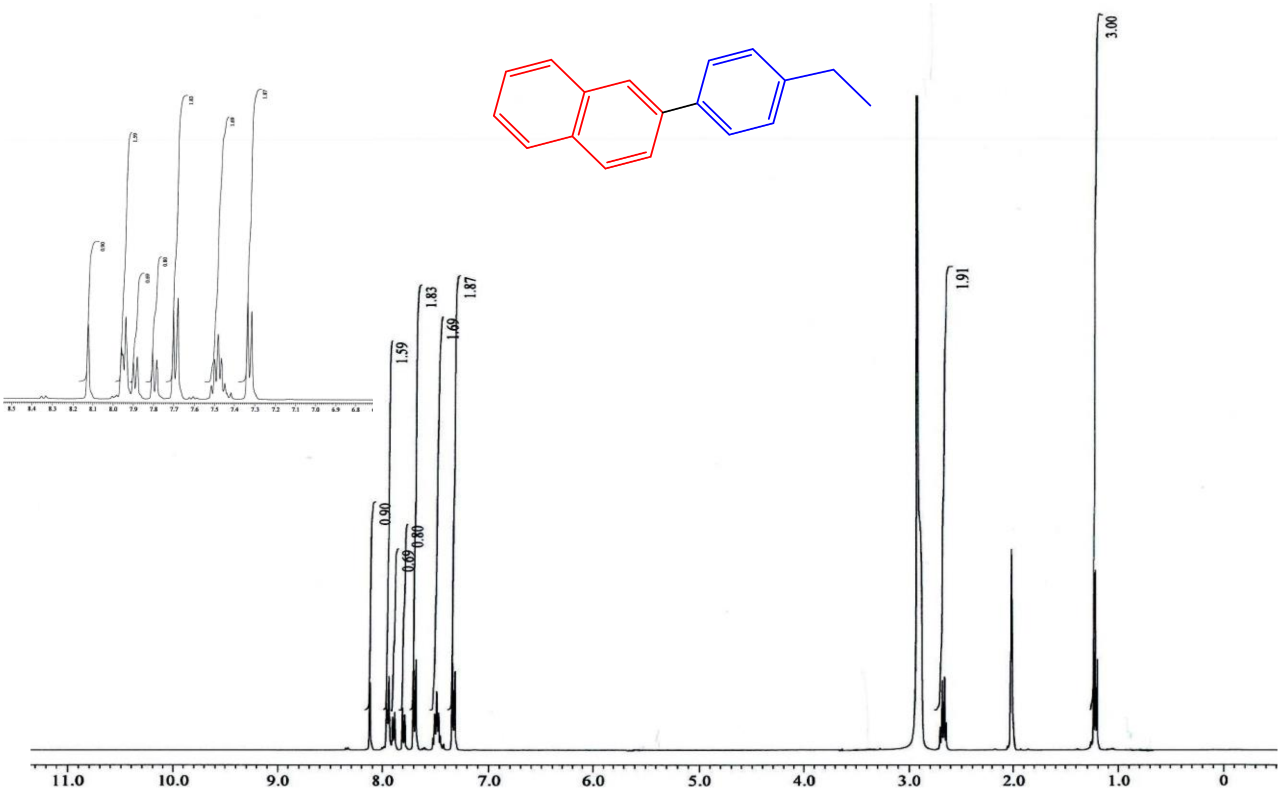

^{13}C NMR:

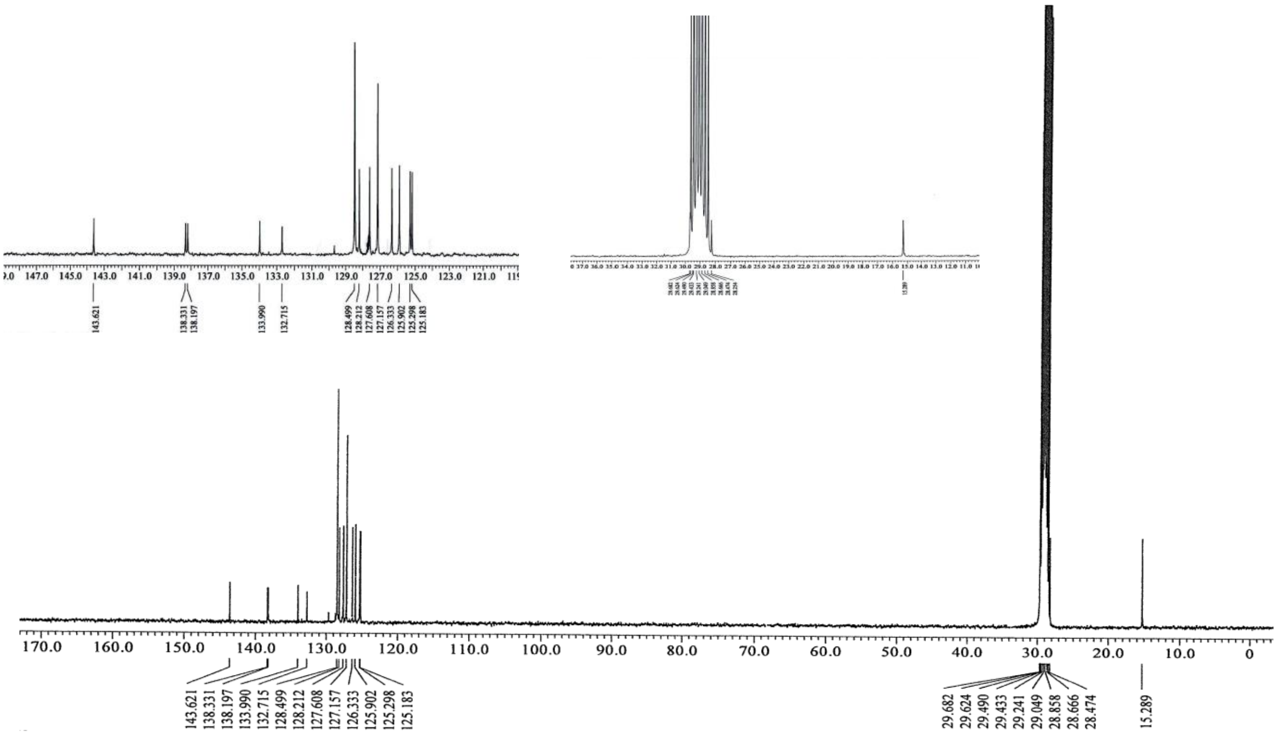

1h: 3-(4-methoxyphenyl)pyridine ^{1}H NMR

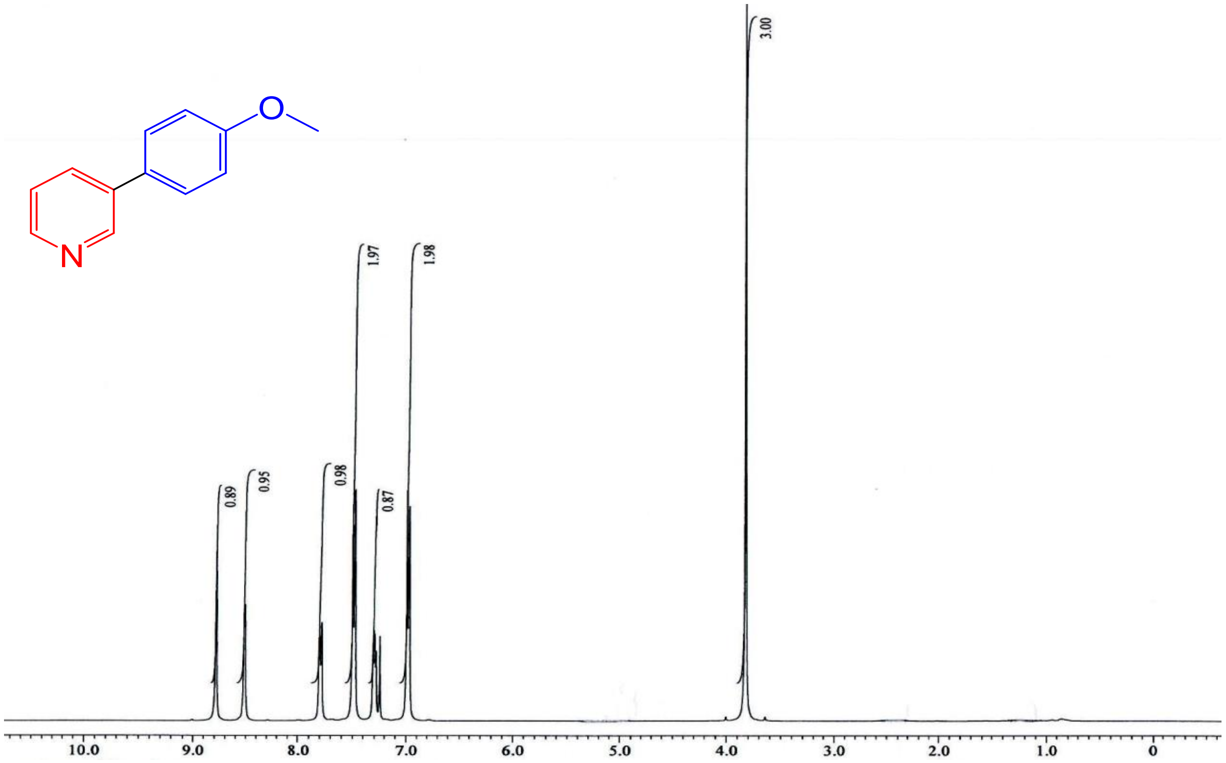

^{13}C NMR:

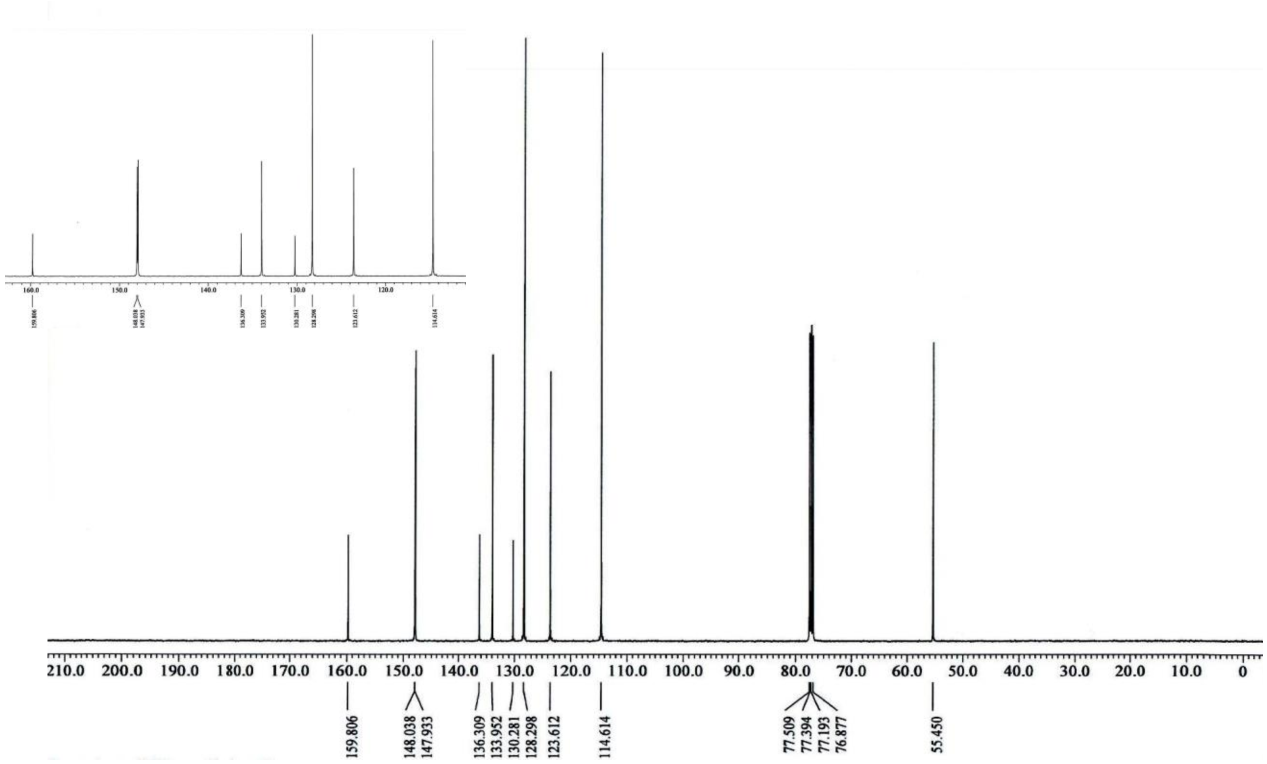

1i: 5-chloro-2-(4-methoxyphenyl)pyrimidine ^{1}H NMR

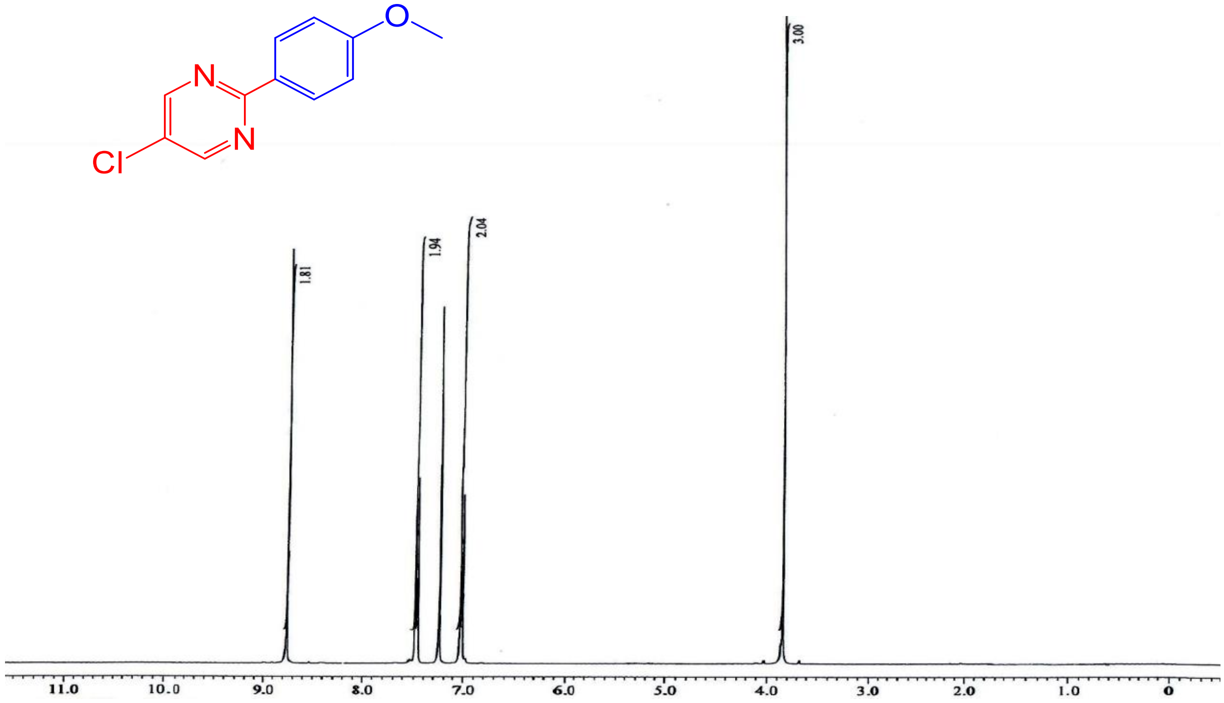

^{13}C NMR:

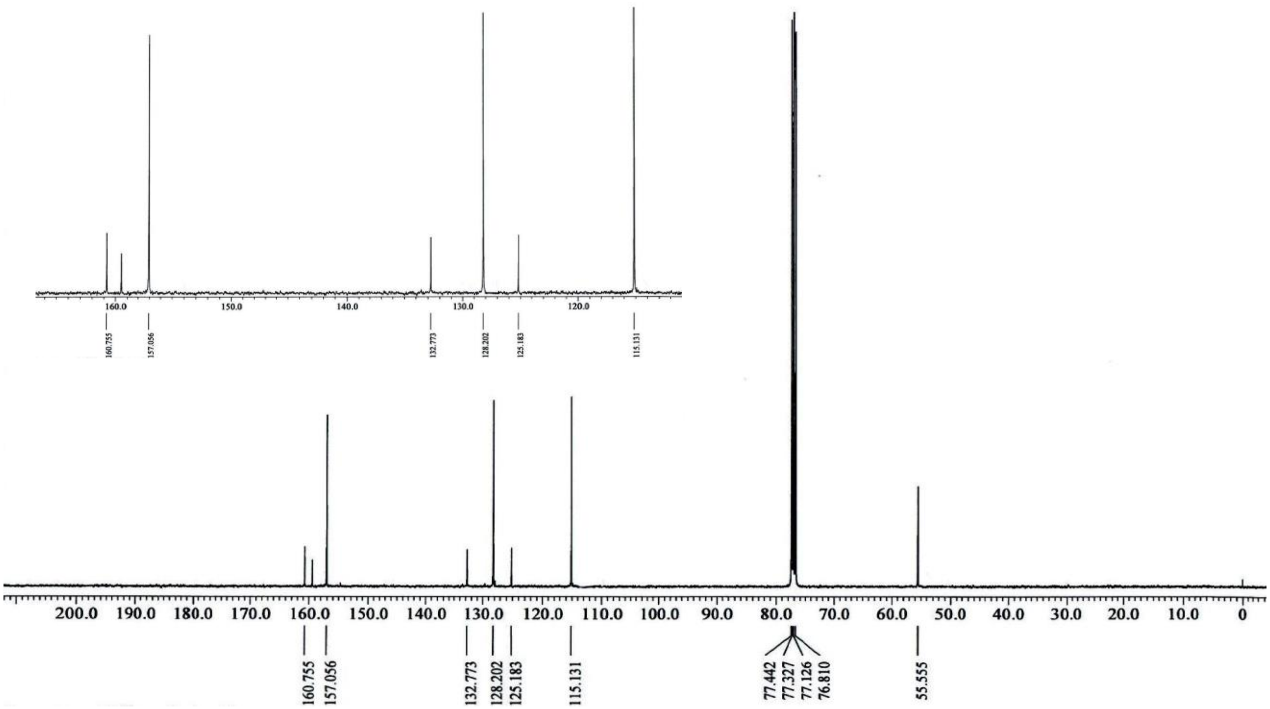

Supplement: Supplementary file 1 — ie4c02922_si_001.pdf [file ie4c02922_si_001.pdf]
